# Gene expression regulation by the Chromodomain helicase DNA-binding protein 9 (CHD9) chromatin remodeler is dispensable for murine development

**DOI:** 10.1371/journal.pone.0233394

**Published:** 2020-05-26

**Authors:** Andrej Alendar, Jan-Paul Lambooij, Rajith Bhaskaran, Cesare Lancini, Ji-Ying Song, Huub van Vugt, Margriet Snoek, Anton Berns

**Affiliations:** 1 Division of Molecular Genetics, The Netherlands Cancer Institute, Amsterdam, The Netherlands; 2 Department of Experimental Animal Pathology, The Netherlands Cancer Institute, Amsterdam, The Netherlands; Ohio State University, UNITED STATES

## Abstract

Chromodomain helicase DNA-binding (CHD) chromatin remodelers regulate transcription and DNA repair. They govern cell-fate decisions during embryonic development and are often deregulated in human pathologies. *Chd1-8* show upon germline disruption pronounced, often developmental lethal phenotypes. Here we show that contrary to *Chd1-8* disruption, *Chd9*^*–/–*^animals are viable, fertile and display no developmental defects or disease predisposition. Germline deletion of *Chd9* only moderately affects gene expression in tissues and derived cells, whereas acute depletion in human cancer cells elicits more robust changes suggesting that CHD9 is a highly context-dependent chromatin regulator that, surprisingly, is dispensable for mouse development.

## Introduction

Normal organismal development requires accurate transcriptional control. This is achieved by myriad of transcription factors and chromatin regulators that act in concert to control expression of the right genes at the right time. Chromodomain helicase DNA-binding enzymes belong to the superfamily of chromatin remodelers that control nucleosomal structure and phasing to regulate gene expression and DNA repair. They contain hallmark amino-terminal double chromodomains and central SNF2 helicase-like ATPase domains, allowing nucleosome recognition and remodeling [1]. Different accessory domains facilitate these actions [1]. The CHD enzymes have limited DNA-specificity and rely on specific histone modifications and transcription factors for recruitment to target loci [2]. Based on their constituent domains, they are classified into three subfamilies: subfamily I (CHD1 and CHD2), subfamily II (CHD3, CHD4 and CHD5) and subfamily III (CHD6, CHD7, CHD8 and CHD9).

In addition to the hallmark domains, members of the subfamily III contain SANT–like and tandem brahma kismet domains (BRK), implicated in DNA [3] and CTCF [4] binding, respectively. These enzymes are homologs of *Drosophila* Kismet (KIS-L), a *Trithorax* family protein that counteracts gene repression by *Polycomb* group proteins during development [5]. It regulates early stages of RNA Polymerase II elongation [6] and is involved in key developmental signaling pathways [7]. Importantly, *kismet* loss-of-function causes specific segmentation defects and homeotic transformation [8] resulting in lethality. Most of these functions have been conserved in the mammalian homologs.

Subfamily III members are found in both promoter and enhancer regions, regulating different stages of RNA Polymerase I and II transcription [2,9]. Subfamily III members have limited DNA affinity [10], and their recruitment to target loci is mediated through interaction with methylated histone tails [9,11] and transcription factors [2]. Despite their high sequence similarity, these enzymes display different substrate specificities and remodeling activities *in vitro* [9,10].

CHD enzymes appear to have non-redundant functions in murine embryonic development [12]. *Chd7* knockout animals survive up to embryonic day 10.5 (E10.5) [13], whereas homozygous deletion of *Chd8* is embryonic lethal at E5.5–7.5 due to widespread p53-mediated apoptosis [14]. These enzymes act in a dose-dependent manner and mice heterozygous for *Chd7* or *Chd8* faithfully recapitulate features of human CHARGE syndrome [15] and autism spectrum disorders (ASD) [16], respectively.

Subfamily III enzymes are often deregulated or mutated in human cancers. In aggressive breast cancers, *CHD7* amplification and overexpression correlates with poor prognosis. In contrast, heterozygous loss of *CHD9* is observed in 55% of breast cancer patients [17]. Low expression is also associated with poor prognosis in colorectal cancer [18]. In addition, a number of studies reported frequent mutations in human cancers: *CHD6* in bladder cancer [19], *CHD7* in medulloblastoma [20,21], whereas *CHD8* harbors mutations in many cancer types [22].

CHD9 (also known as PRIC320 and CReMM) is the least characterized member of the subfamily. It was identified as transcriptional regulator in rat liver [23] and mesenchymal stem cells [24]. Biochemical studies revealed CHD9 interaction with H3K4me2/3, H3K9me2/3 and H3K27me3 modified histones and illustrated its capacity to reposition nucleosomes in an ATP-dependent manner [9].

CHD9 associates with nuclear receptors and contributes to glucocorticoid receptor–mediated gene expression regulation [23,25] and might be involved in loosening the chromatin structure in mouse oocytes [26]. Recent landmark study in mouse ES cells revealed that CHD9 chromatin occupancy correlates with gene expression levels and the presence of the H3K4me3 mark at a subset of loci [27].

While these studies address biochemical and functional properties of CHD9, the *in vivo* functions of CHD9 have remained unresolved. Here, we describe the first *Chd9* knockout mice and study its contribution to development and lymphomagenesis, a tumor subtype in which insertional mutagenesis experiments suggested a potential role of CHD9.

## Results

### CHD9 is dispensable for murine development and knockout does not display overt phenotypic aberrations

*Chd9* is ubiquitously expressed in murine tissues with the highest levels observed in brain [23]. Previous studies implicated CHD9 in stem cell biology [28], osteogenesis [29] and steroid-hormone signaling [23,25], whereas insertional mutagenesis screens suggested an oncogenic driver role in murine [30,31] and human lymphomagenesis [32]. To explore the contribution of CHD9 to development and tumorigenesis, we generated *Chd9* knockout mice.

**Fig 1:**
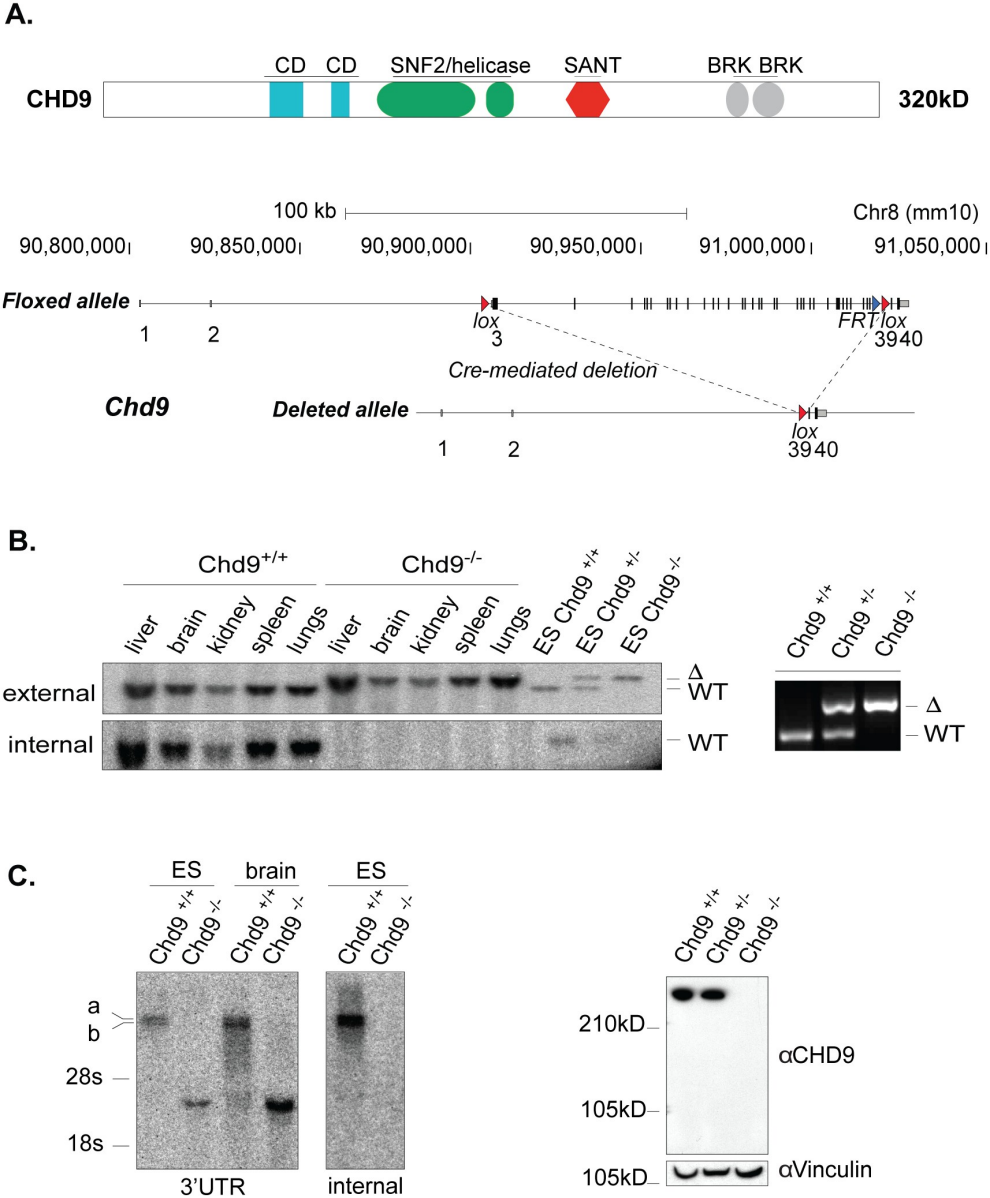
Generation of the *Chd9* knockout results in complete removal of the locus coding potential. (A) (Above) Representation of the CHD9 protein domains- CD (chromodomain), BRK (Brahma-Kismet domain), and (bellow) targeting strategy whereby *Cre*–mediated deletion removed coding potential of the locus. (B) Left panel, Southern blot analysis of genomic DNA from adult organs and ES cells from wild type and *Chd9* knockout animals, using external and internal radioactive probes. Right panel, PCR detection of deleted allele using primers 117kb apart. (C) Left panel, Northern blot mRNA detection in adult brain and ES cells using 3’UTR and internal cDNA PCR–probes. Right panel, Western blot analysis showing complete depletion of the CHD9 protein in ES cells. Vinculin used as a loading control.

**Fig 2:**
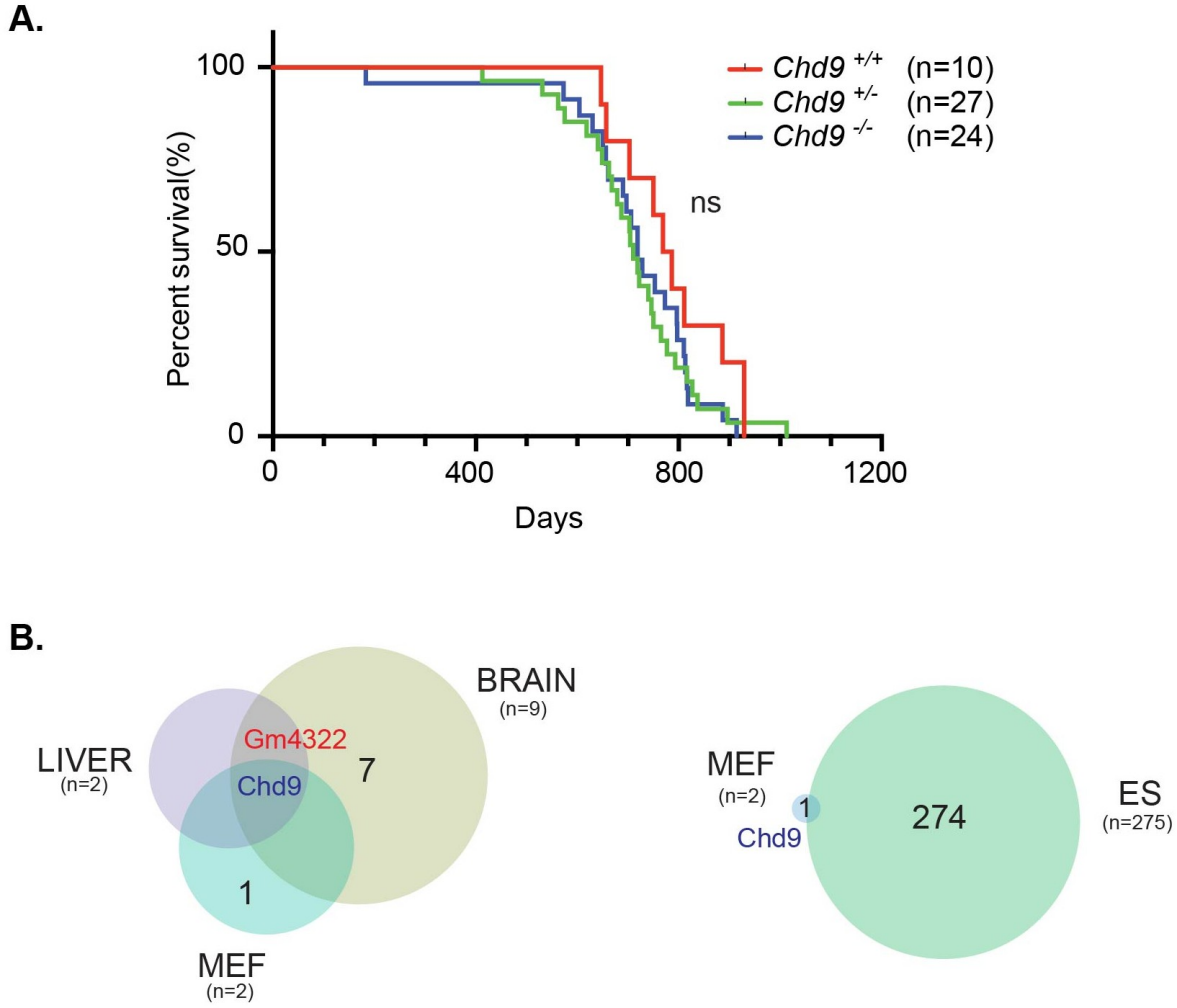
*Chd9* deletion does not affect murine survival and has moderate effect on gene expression. (A) *Chd9* knockout animals are viable, fertile and display no overt phenotypes. Kaplan–Meier survival curves reveal comparable life expectancies of wild-type, heterozygous and homozygous knockout animals (ns = not significant, log-rank test p–value = 0.272). (B) Venn diagram representation of the overlap between differentially expressed genes (DEGs) identified in *Chd9*^–/–^MEFs, ESCs, E15 brain and liver. Numbers in parenthesis represent total number of DEGs between *Chd9*^–/–^and *Chd9*^+/+^ cells and organs (p-adj < 0.05, log_2_FC (fold change) > ±1). The numbers in black indicate the number of non-overlapping genes between analyzed groups.

The vast majority of *Chd* family knockouts are embryonic lethal [12,33,34]. The reported allele targeting strategies for *Chd1-8* employed gene-trap reporter constructs, the deletion of critical exons or the removal of functional domains ([12] and references therein). In the case of *Chd9* this was a particular challenge. *Chd9*, encoding a protein of 320 kD with a diversity of functional domains (Fig 1A), contains several translation-initiation sites downstream from the first ATG codon, raising the possibility of translation of shorter, truncated protein isoforms that might exhibit dominant-active or dominant-negative functions. To circumvent this, we deleted all protein coding exons of the locus by flanking the *Chd9* open reading frame (ORF) with two *loxP* sites by homologous recombination in mouse ES cells (ESC)(Fig 1A, S1 Fig). Successful gene targeting of *LoxP* sites to the same *Chd9* allele was confirmed by Southern blot and PCR. The resulting *Chd9* floxed mice (*Chd9*^*f/+*^) were crossed to the β-actin-driven Cre-recombinase strain (*ACTB-Cre*) to obtain heterozygous *Chd9* (*Chd9*^*+/–*^) animals (Fig 1A) and backcrossed to FVB for two generations. *Chd9*^*+/–*^males and females were mated and offspring characterized. Homozygous *Chd9*^*–/–*^animals were born at Mendelian ratio’s, appeared viable and fertile with no immediate obvious defects (Fig 2A). The germline-deletion of *Chd9* also did not affect lifespan (log-rank test, p–value = 0.272; Fig 2A). This was striking, as all hitherto published *Chd* family knockouts display severe phenotypes, ranging from embryonic lethality [12,33,34] to male infertility [35].

Southern blot and PCR analysis of tissues and primary cells derived from wildtype, heterozygous, and homozygous littermates showed complete deletion of the locus (Fig 1B). The *Chd9* gene encodes two mRNA isoforms (Fig 1C and S6A Fig). Western blot and immunofluorescence staining confirmed depletion of the CHD9 protein in derived *Chd9*^*–/–*^ESCs and murine embryonic fibroblasts (MEFs) (Fig 1C, S2 Fig). Deletion of the ORF resulted in a shorter spliced mRNA species that corresponds to the remaining *Chd9* exons that do not encode any CHD9-specific protein domains (Fig 1C, S6A Fig).

Histopathology on small cohorts of matched littermates (3 months; wildtype (n = 5), homozygous knockout (n = 5)) and older (12 months; wildtype (n = 6), homozygous knockout (n = 6)) revealed no significant pathological changes, with mild inflammation in the liver, lung, kidney and Harderian gland being the most distinctive (S1 Table). Wildtype, heterozygous and homozygous littermates, that were aged and examined when moribund, displayed only the phenotypic features characteristic for the strain background or old age. The majority of these animals were older than 18 months when analyzed (Fig 2A). The most encountered tumor types were adenocarcinomas/papillary carcinomas of the lung and some hepatomas, adenomas of the Harderian gland, adenomas of the pituitary gland, adenocarcinomas of the mammary gland, squamous cell carcinoma of the mucosal lining of the oral cavity and vagina, and a few sarcomas such as histiocytic sarcoma, leiomyosarcoma and lymphoma (S1 Table). The incidence and the type of the neoplasia were rather evenly distributed among the different genotypes. Therefore, the pathologies found in the *Chd9*^*–/–*^mice were likely due to old age and the strain genetic background. Collectively, the survival and histopathological analysis indicate that *Chd9* knockout animals are viable, fertile and display no overt phenotypes.

### CHD9 depletion has a moderate effect on gene expression during murine embryogenesis

Absence of overt phenotypes in *Chd9*^*–/–*^mice could be due to activation of compensatory transcriptional programs or functional redundancy with other CHD enzymes. Additionally, CHD9 might exert its functions during embryonic development rather than in adult tissues with a concomitant greater potential for compensatory mechanisms to kick in. In adult mice *Chd9* is ubiquitously expressed, with the highest levels in brain (our data and [23]). During murine embryogenesis, we observed high *Chd9* mRNA levels at E13.5 and E15 (S8 Fig). High expression was also observed in derived MEFs and ESCs (Fig 1C, S8 Fig). To explore the role of CHD9 in gene expression regulation during development, we performed gene expression analyses of knockout tissues (E15 liver and brain) and derived primary cells.

RNA–seq of *Chd9* knockout embryonic (E15) liver and brain revealed only a handful of differentially expressed genes (DEGs) (adjusted p–value≤ 0.05, log_2_FC ≥ ±1) (Fig 2B, S3 Fig). Importantly, *Chd9* was the only downregulated mRNA in both tissues (Fig 2B, S3 Fig). Moreover, both liver and brain show concordant upregulation of an uncharacterized pseudogene *Gm4322* (Fig 2B, S3 Fig). Despite high *Chd9* expression in the developing brain (S3B Fig), only marginal upregulation of the *Hoxa* (a7), *Hoxb* (b6, b7, b9) and *Hoxc* (c5, c6) homeotic gene clusters was observed (S3B Fig). Gene-set enrichment analysis (GSEA) of *Chd9* knockout brain revealed global downregulation of genes involved in complement and coagulation cascades (KEGG: hsa04610), recently emerging as important regulators of the developing brain [36] (S12 Fig). This might point to underlying transcriptional perturbations in embryonic brain in the absence of CHD9, albeit without overt phenotypic consequences. In the knockout embryonic liver, GSEA showed upregulation of genes involved in Peroxisome functioning (KEGG: hsa04146) (S12 Fig), in agreement with its previously identified role as peroxisome proliferator–activated receptor α (PPARα) interactor in liver [23].

RNA-seq analysis of knockout MEFs revealed only downregulation of *Chd9* (Fig 2B) and upregulation of the high mobility group protein B2 (*Hmgb2*) pseudogene *Gm13237* (S4A Fig). Pseudogenes might function as competitive endogenous RNAs (ceRNAs) regulating parent gene expression [37]. However, Western blot analysis revealed no difference in protein levels or isoform expression of HMGB2 and related HMGB1 proteins in *Chd9*^–/–^MEFs (S5 Fig).

Conversely, in derived *Chd9*^–/–^ESCs maintained in serum–free 2i media with leukemia inhibitory factor (LIF), more profound gene expression changes were seen. In total, 63 upregulated and 212 downregulated genes were observed (Fig 2B, S4B Fig). The GSEA revealed downregulation of hedgehog signaling (HH) (KEGG: hsa04340) in knockout ESCs (S12 Fig), implicating *Chd9* as putative positive regulator of HH signaling. Notably, in *D*.*melanogaster*, KIS-L is a transcriptional repressor of hedgehog signaling required for proper wing development [7]. The absence of gene expression changes in *Chd9*^–/–^E15 tissues and MEFs appears not to be due to compensatory transcriptional deregulation of other CHD enzymes or Polycomb repressive complex 1 and 2 proteins (PRC1 and PRC2) (S3 and S4 Figs).

### *Chd9* knockout mice display a mild expansion of the short-term hematopoietic stem cell compartment

Of the CHD subfamily III, CHD7 regulates developmental hematopoiesis [38] and expansion of hematopoietic stem and progenitor cells, whereas CHD8 is required for survival of both non-transformed pre-B cells and acute lymphoblastic B-cell leukemia cells as well as *EμMyc* B-cell lymphomas [39]. In view of the role of these subfamily III CHDs in normal and malignant hematopoiesis, we asked whether *Chd9* germline deletion might perturb hematopoiesis.

**Fig 3:**
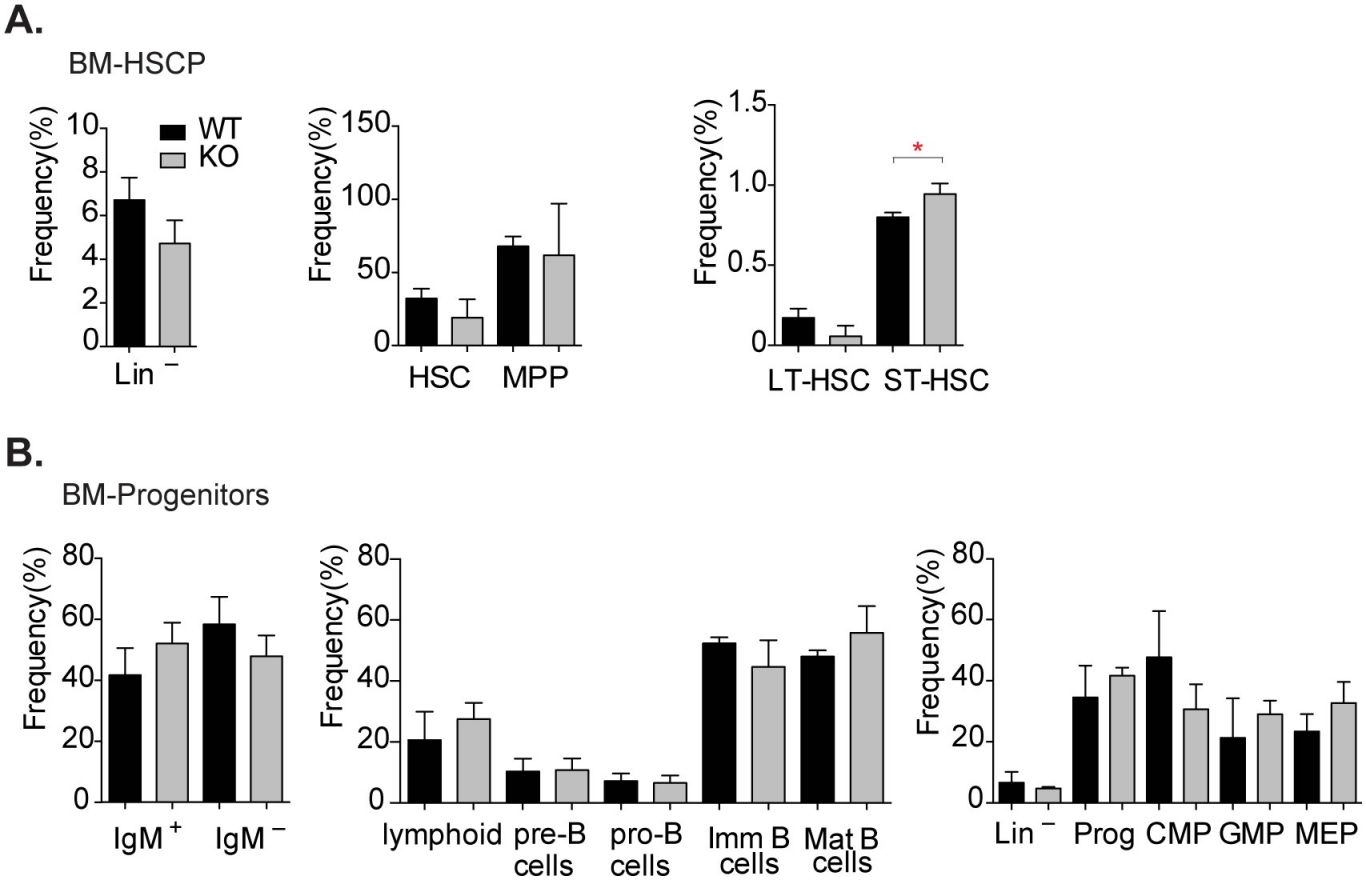
*Chd9* knockout animals display mild effect on hematopoietic stem cells. (A,B) Flow cytometric analysis of *Chd9* wild-type (WT, n = 3) and knockout (KO, n = 4) bone marrow (BM) stem cell and progenitor cells revealed expansion of short–term hematopoietic stem cells (red asterisk denotes p–value: 0.018, ST–HSC). Other BM hematopoietic stem cell and progenitor populations display no statistically significant differences. Data plotted as frequency of hematopoietic cells, mean ±SD, p-value calculated using two-tailed Student’s t–test. HSCP- hematopoietic stem cells and progenitors: Lin−(lineage negative), HSC (hematopoietic stem cells), MPP (multipotent progenitor cells), LT-HSC (long-term hematopoietic stem cells). Progenitors: IgM^+^ (B-cell progenitors), CMP (common myeloid precursor), GMP (granulocyte-monocyte progenitors), MEP (megakaryocyte-erythroid progenitors).

Flow cytometric analysis (FACS) of bone marrow (BM) of *Chd9*^*–/–*^mice showed a reduction in the lineage-negative (Lin^-^) adult hematopoietic stem and progenitor cell population (HSPCs), although this was not statistically significant (p-value = 0.055), (Fig 3A). We also observed a tendency for a reduced frequency of the hematopoietic stem cells (HSCs, p–value = 0.53) and multipotent progenitors (MPPs, p–value = 0.78). Notably, *Chd9*^*–/–*^animals displayed a reduced frequency of the long-term hematopoietic stem cells (LT-HSC, p–value = 0.06) and statistically significant expansion of short-term hematopoietic stem cell (ST-HSC, p–value = 0.02) (Fig 3A). Depletion of CHD9 might affect cell–intrinsic programs (transcriptional or metabolic) or interaction with local stem cell niches, impeding self–renewal of LT-HSCs. In turn, this may lead to a compensatory increase of the ST-HSCs compartment, thereby maintaining hematopoietic homeostasis [40]. However, there was no significant effect on frequency and number of bone marrow multipotent progenitors (MPPs), common lymphoid (CLP) and common myeloid progenitors (CMP), and their more differentiated progenitors (Fig 3B). Likewise, *Chd9*^*–/–*^and control animals displayed comparable frequencies of thymic and splenic lymphoid cells (S6A and S6B Fig), as well as mononuclear leukocytes (lymphocytes, monocytes and macrophages) and granulocytes (eosinophils and neutrophils) in peripheral blood (S6C Fig). Taken together, FACS analysis revealed that *Chd9*^*–/–*^mice display a modest expansion of ST–HSC in bone marrow, with no discernible effects on lineage–restricted progenitors and white blood cells.

### CHD9 does not contribute to EμMyc -driven lymphomagenesis or transformation of MEFs

**Fig 4:**
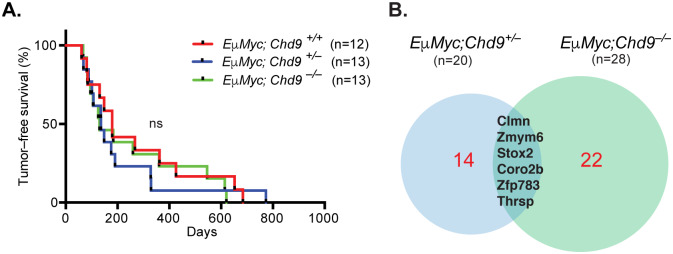
*Chd9* deletion does not affect latency or gene expression of *EμMyc*–driven lymphomagenesis. (A) Kaplan–Meier plot shows comparable tumor-free survival of *EμMyc;Chd9* animals (ns = not significant, log-rank test, p–value = 0.78). (B) Venn diagram representation of the overlap between differentially expressed genes (DEGs) identified in *EμMyc;Chd9*^+/–^(left) and *EμMyc;Chd9*^–/–^(right) compared to control *EμMyc;Chd9*^+/+^ lymphomas. Numbers in parenthesis represent total number of DEGs (p-adj < 0.05, log_2_FC (fold change) > ±1). The DEGs common for *EμMyc;Chd9*^+/–^and *EμMyc;Chd9*^+/+^ samples are highlighted in black and non-overlapping genes in red.

Several studies point to a role of CHD subfamily III members as drivers of hematopoietic cancers. CHD8 is required for survival of pre-B cells, acute lymphoblastic B-cell leukemia cells, and *EμMyc* B-cell lymphomas [39]. Interestingly, insertional mutagenesis screens, performed by us and others, identified the *Chd9* locus as common integration sites in murine [30,31] and human lymphomagenesis [32]. Moreover, downregulation of *CHD9* expression is reported in multiple myeloma [41] and diffuse large B–cell (DLBCL) lymphoma patients [42]. To more rigorously document a potential role of CHD9 in lymphomagenesis, we crossed the *Chd9*^*–/–*^strain with *EμMyc* transgenic mice hypothesizing that if CHD9 would have an oncogenic or tumor-suppressing activity this should be revealed through an acceleration or delay of tumorigenesis, respectively. However, *EμMyc-*driven lymphomagenesis was not affected by the *Chd9* status (ns = not significant, log-rank test, p–value = 0.78) (Fig 4A) and the observed lesions showed the typical marker profile well known from *EμMyc* mice (S7B Fig).

In order to assess whether CHD9 showed an effect on lymphoma gene expression, we profiled lymphomas from *EμMyc Chd9* wildtype, heterozygous and knockout mice by RNA-seq. In the heterozygous tumours, 20 genes were upregulated and only leucine rich repeat neuronal 4 (*Lrnn4*) downregulation was observed, whereas in the knockout tumours 28 genes were up–and 8 down–regulated (S7C Fig). The presence of a shared subset of upregulated genes in both CHD9 heterozygous and knockout lymphomas suggests that they might be direct target genes (Fig 4B, S7C Fig) whereas the modest changes in gene expression observed between tumors likely reflect variability in collaborating lesions elicited during tumor development.

Since *Chd9* is highly expressed in MEFs, we also examined its requirement for cell proliferation and transformation. *Chd9* deletion did not affect proliferation under normal culture conditions, or anchorage-independent growth of RasV12/Myc–or RasV12/E1a–transformed MEFs (S9 Fig).

These results indicate that *Chd9* deletion does not affect latency or histopathological features of *EμMyc*-driven lymphomagenesis nor does it modulate the oncogenic transformation of MEFs.

### CHD9 depletion deregulates gene expression in human cancer cell lines

**Fig 5:**
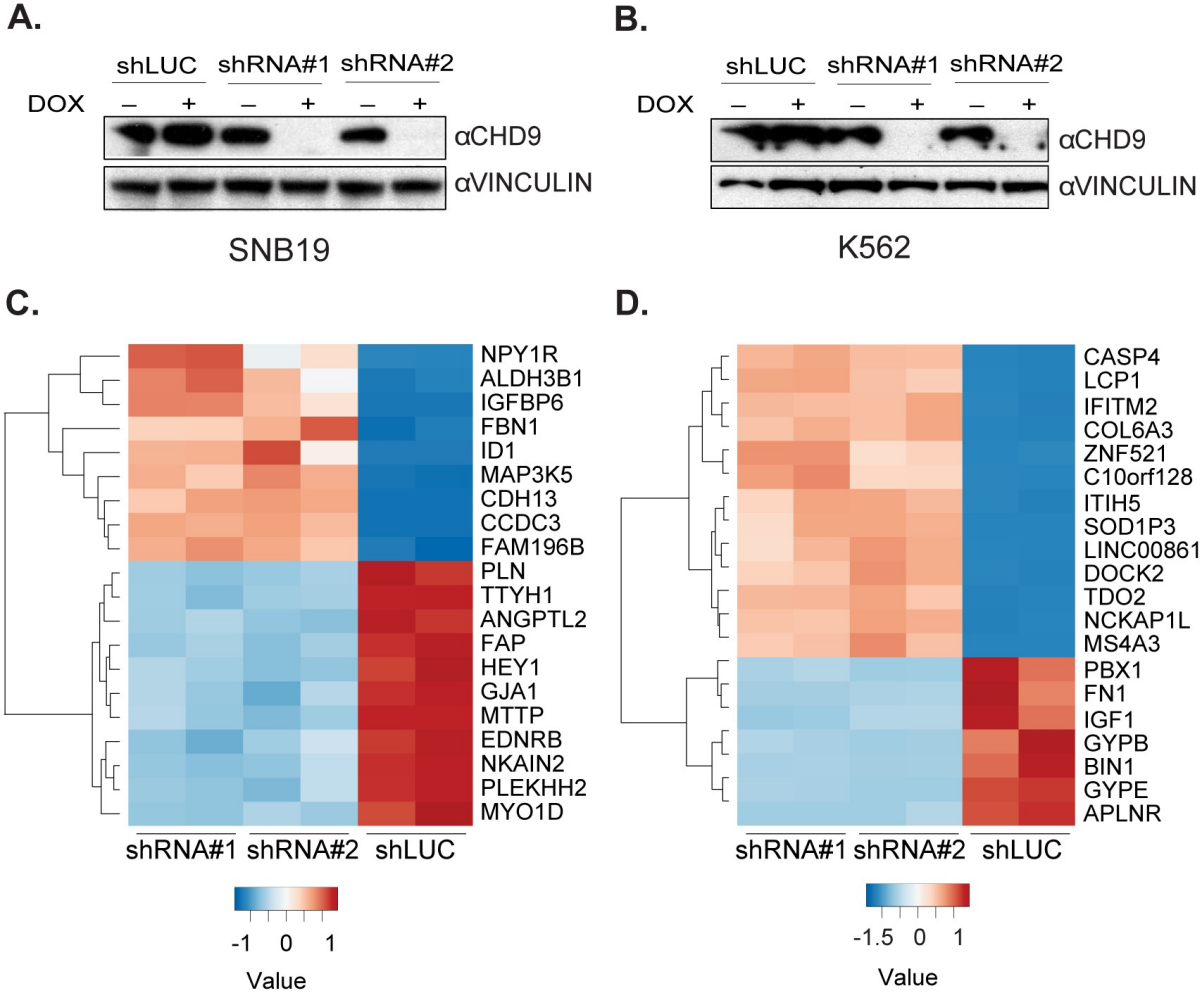
CHD9 depletion deregulates gene expression in human SNB19 and K562 cancer cells. (A, B) Western blots reveal depletion of the CHD9 protein by RNA-interference 48-hrs post doxycycline addition (±DOX). (C, D) Heatmaps of the top 20 most significant DEGs based on the p-adj value in SNB19 and K562 cells upon CHD9 knockdown. The color scale is based on the normalized read count values.

Lastly, we explored gene expression regulation by CHD9 in human cancer cell lines. Publicly available data sets indicate that CHD9 is overexpressed in cancers of neuronal and hematopoietic origin (S10 Fig). Analysis of a panel of human cancer cell lines revealed high CHD9 protein levels in SNB19 glioblastoma and K562 chronic myelogenous leukemia cell lines (Fig 5A and 5B, S8 Fig). To address the role of CHD9 in gene expression regulation in these human cancer cell lines, we generated SNB19 and K562 cell lines bearing doxycycline-inducible short hairpin RNAs (shRNA) targeting *CHD9* and confirmed successful depletion of the protein upon knockdown induction by Western blotting (Fig 5A and 5B). Contrary to observations in *Chd9*^*–/–*^mouse models, RNA-seq analysis in these cell lines revealed profound gene expression changes 48 hours upon CHD9 depletion (Fig 5C and 5D, S11 Fig). In K562 cells, *CHD9* knockdown resulted in 215 down- and 635 upregulated genes, whereas in SNB19 cells, 121 down- and 113 up–regulated genes were observed (S11 Fig). The GSEA revealed global downregulation of genes involved in oxidative phosphorylation (KEGG: hsa00190) in K562 cells, whereas in SNB19 the analysis showed downregulation of genes involved in cerebellar long-term depression (KEGG: hsa04730), considered a molecular basis for cerebellar learning (S12 Fig). Notably, a number of deregulated genes in CHD9-depleted K562 cells–such as *PBX Homeobox1* (*PBX1)*, *Apelin receptor (APLNR)* and *Bridging Integrator 1 (BIN1)*–are implicated in neuronal development and pathology [43]. This suggests a more widespread role for CHD9 in regulation of neuronal-specific genes, which even shows up in the context of malignant hematopoietic K562 cells. We did not find significant overlap in DEGs between both human cancer cell lines. This was not unexpected, as K562 and SNB19 cells represent different lineages and CHD9 may exert context–dependent roles as a repressor or activator of gene expression. Interestingly, despite robust changes in gene expression, we did not observe differences in proliferation upon depletion of CHD9 in these cells (S11 Fig) in line with recent genome-wide CRISPR-Cas9 drop-out screens that failed to identify essential roles for CHD9 in a comprehensive panel of human cancer cell lines [44].

## Discussion

Our results reveal that, despite ubiquitous expression, *Chd9* is dispensable for embryonic development. Furthermore, knockout animals are viable, fertile, display no overt phenotypes and have a normal lifespan. We have only observed modest expansion of ST-HSC in bone marrow, however, without discernible effect on other progenitors and mature populations. Moreover, although several murine and human studies suggested a role for *Chd9* in solid tumors and lymphomas, CHD9 depletion did neither accelerate nor impair *EμMyc*-driven lymphomagenesis. Likewise, CHD9 is not required for proliferation or oncogenic transformation of primary MEFs. Nevertheless, a complex protein of 320 kD with multiple functional domains and highly conserved in evolution should have an important role. The lack of phenotype we noted is especially remarkable given the pronounced phenotype of nearly all other CHD proteins. However, if the role of CHD9 has evolved during evolution for specific neuronal functions, that are not required or do not become apparent in the routine monitoring taking place in dedicated mouse facilities, it might be not too surprising that our analyses did not reveal such functions. Future detailed studies will be required to elucidate this.

## Materials and methods

### Animal study design and ethics statement

The study was performed in accordance with the Dutch and European regulations on care and protection of laboratory animals. All animal experiments were approved by the local Animal Ethics Committee—Dierexperimentencommissie (DEC) of The Netherlands Cancer Institute (NKI)- (OZP ID: 05029). All animal strains used in this study were generated or obtained through The Netherlands Cancer Institute Animal Facility. The number of animals required for each of the analyses performed in this paper are indicated either in the main text, figure panels or figure legends. The comparison of survival curves between experimental animal cohorts is done using log-rank (Mantel-Cox) test using GraphPad Prism8. For all calculations of p-values, significance threshold alpha was set at 5% (p< 0.05).

Since no previous knowledge on CHD9 role in murine development/lifespan is available, duration of the experiment was set for the maximum period of three years as to be able to show an effect on longevity in case this would be influenced by CHD9. Previous studies show that *EμMyc* mice succumb to lymphomas at average of 5 months. *EμMyc;Chd9* mice were monitored for the maximum period of two years, as it was not known whether *Chd9* loss would accelerate or decelerate tumorigenesis.

Animals were kept in standard husbandry conditions (open-cages, standard condition of bedding and enrichment, feeding, light, and temperature, with free access to food and water). Animals were monitored daily by trained animal technicians at the NKI Animal Facility. Any symptoms of disease or poor health (weight loss, abnormal sedentary behaviour, difficulty breathing, uncoordinated or impaired movement) were considered grounds for euthanasia. Moribund animals were promptly euthanised with CO_2_, confirmed by lack of response to firm toe pinch, and necropsy was performed to identify macroscopic changes and, when deemed needed, tissue samples were processed for immunohistopathology to document microscopic abnormalities. No animals died prior to meeting the criteria for euthanasia. No anaesthesia, analgesia or special techniques were required or applied during the experiments.

### Generation of the Chd9 conditional knockout mouse strain

The endogenous *Chd9* locus on chromosome 8 was flanked by *loxP* sites on both sites of the gene in order to excise the whole coding potential of the locus upon *Cre*-mediated recombination. The 5’ *loxP* site was introduced upstream of the exon 3 (containing ATG translation initiation site), whereas at the 3’ end a second *loxP* site was introduced downstream of the exon 38. Targeting was performed in E14 ES IB10 cells of 129P2 genotype using the recombineering-based method for generating conditional knockout mutations [45]. Since ES cells were targeted at two sites 117 kb apart, the original method had to be adapted for two-step targeting strategy.

#### Construction of retrieval and targeting vectors

Primer sequences used for constructing the *Chd9* conditional knockout vector are listed below:

5’ primer A NotI-Chd9 5’ ATAAGCGGCCGCGTGAAAGCAAGCGGCTTA

5’ primer B HIII-Chd9 5’ GTCAAGCTTCTGGGTGTGGTGATGTACG

5’ primer Y HIII-Chd9 5’ GTCAAGCTTGCGGTGTTATAGAATATGAGCG

5’ primer Z SpeI-Chd9 5’ TCTACTAGTGAATCACTTGGAAACACTCG

5’ primer C NotI-Chd9 5’ ATAAGCGGCCGCCACAGAGAAACCCTGTCTGA

5’ primer D RI/BglII-Chd9 5’ GTCGAATTCAGATCTCAAGCATCTTCCTATAGTCAG

5’ primer E BamHIII-Chd9 5’ ATAGGATCCCATTCGCTTCACAGATACCT

5’ primer F SalI-Chd9 5’ CGCGTCGACGTCGCGGTAGGTTATTACGGTATCA

3’ primer A NotI-Chd9 5’ ATAAGCGGCCGCATGAACTGATCCTCGGTTACA

3’ primer B HIII-Chd9 5’ GTCAAGCTTACGCCAAATGGAGTATACG

3’ primer Y HIII-Chd9 5’ GTCAAGCTTGAAAGGCTATCCCAGTACGT

3’ primer Z SpeI-Chd9 5’ TCTACTAGTACACCAAGACCAACATCATCCG

3’ primer C NotI-Chd9 5’ ATAAGCGGCCGCTCCCTGTCTTGCCTCCTATG

3’ primer D RI/BglII-Chd9 5’ GTCGAATTCAGATCTATTGCTAATTAACGTAGATGTCG

3’ primer E BamHI-Chd9 5’ ATAGGATCCCAAGACGGTCCTTCCATGG

3’ primer F SalI-Chd9 5’ CGCGTCGACGTCCAATCTGCAACAATATGCGTA

The 5’ arm was retrieved from EL350 cells from BAC RP23-296 (RPCI-BAC library, de Jong lab) [46]. The 3’ arm was retrieved from EL350 cells from BAC bMQ-244 (129 S7 Geneservice, Cambridge, UK).

The retrieval vector was generated by mixing 3 μL of PCR product 1 (left arm AB, *Not*I/*Hin*dIII), 3 μL of PCR product 2 (right arm YZ, *Hin*dIII/*Spe*I), 2 μL of *MC1TK*(PL253, *Not*I/*Spe*I), 1 μL of 10× ligation buffer, and 1 μL of T4 DNA ligase.

The *Neo*-targeting vector were generated by mixing 3 μL of PCR product 1 (left arm, *Not*I/*Eco*RI, CD), 3 μL of PCR product 2 (right arm, *Bam*HI/*Sal*I, EF), 2 μL of floxed *Neo* cassette (PL452 or PL451,*Eco*RI/*Bam*HI), 1 μL of the retieved Bac DNA fragment *Not*I/*Sal*I), 1.2 μL of 10× ligation buffer, and 1 μL of T4 DNA ligase.

#### Gene targeting in mouse ES cells

*First targeting*. For gene targeting, 25 μg of *NotI*-linearized 5’ *Chd9* cko-targeting vector DNA was electroporated into 10 × 10^6^ ES cells E14 IB10 (129P2 genotype) were grown on feeder MEFs. Transfectants were selected in CM medium (10% fetal calf serum in GMEM with 2 mM L-glutamine) with G418 (200 μg/ml). Correctly targeted clones were identified by Southern blot analysis of a *HindIII* digest genomic DNA with the 5′ probe (primers F22- 5’ATTTGTGCGCATGAACTGC; R431-5’ GTCGAGCAGTAAGGCTAAAGTAA). Correctly 5’ targeted clone was electroporated with the PGK-Cre/PGK-Puro plasmid in order to excise the *loxP* floxed neo cassette. Clones with excised neo cassette were identified by PCR analysis using primers FRW- 5’TCTCAGAGGCTGGTGTTTGTTTC; REV- 5’CACAAGAAGGTGACCCAGAA.

*Second targeting*. The selected 5’ targeted clone was electroporated with 25 μg *NotI*-linearized 3’ *Chd9* cko-targeting vector DNA with a *Frt* floxed neo cassette and a *loxP* sequence in the same orientation as the *loxP* sequence at the 5’ side of the *Chd9* gene. Correctly targeted clones were identified by Southern blot analysis of a *HindIII* digest with the 3′ probe (exon 39 primers FRW- 5’TGTTTGTTTGCTTACAGGTTGG; REV- 5’ TCTAAGGCAGAATCAAATGGTTT).

*Cis targeted clones*. In order to detect targeted ES clones with both 5’ and 3’ *loxP* sequences on the same chromosome (cis targeting), double mutant ES cell clones were electroporated for transient Cre expression by PGK-Cre/PGK-Puro plasmid. Excision of the *Chd9* protein-coding exons 3–38 was demonstrated by PCR using primers (Δ-PCR): FRW-5’ CCCGAGTGTGGGTTTAAATG; REV-5’ CCAATCTAACTGGGCTACCG.

#### Generation of conditional *Chd9* knockout mice

Correctly targeted neomycine-resistant ES clones harbouring two *loxP* sequences on the same chromosome flanking the *Chd9* gene, were injected into C57BL/6 blastocysts, and chimeras were crossed with FVB/N mice to produce heterozygous offspring. The resulting *Chd9* ^*f/+*^ heterozygous and subsequent *Chd9* ^*f/+*^ homozygous mice were viable and fertile and showed a normal lifespan, indicating that the *Chd9* ^*f/+*^ conditional allele is fully functional.

#### Excision of the Frt floxed neo cassette

The neo cassette at the 3’ end of the *Chd9* gene was excised by crossing the mice with the *Flpe* deleter strain (FVB background).

#### Generation of heterozygous *Chd9* knockout mice

To generate heterozygous knockout allele, *Chd9* ^*f/+*^ mice were crossed with *Acre* germline deleter strain (Beta-actin driven expression of Cre recombinase). Mice were backcrossed to FVB background for two generations and *Chd9*^+/-^ males and females were bred to generate experimental cohorts.

#### Generation of *EμMyc; Chd9* strain

*Chd9*^*-/-*^ males were crossed with females bearing *EμMyc* transgene [47] (maintained on FVB/NCrl background) to obtain *Chd9*^*+/-*^; *EμMyc* animals, which were subsequently crossed with *Chd9*^*+/-*^ animals to generate experimental cohorts (1/8 *Chd9* wildtype and homozygous, and 1/4 *Chd9* heterozygous animals bearing *EμMyc* transgene).

### DNA isolation and Southern blotting

DNA was isolated from tails and organs using DirectPCR Lysis Reagent (Viagen Biotech). For Southern blot 10 μg of DNA was digested with 4 μL *HindIII* (Roche) in reaction containing 7 μL buffer B (NEB), 1.4 μL spermidine (Sigma Aldrich) and 0.7 μL BSA(Sigma) in 70 μL volume. DNA was resolved on 0.8% agarose gel at 30V overnight and transferred to Amersham Hybond N^+^ membrane (GE Healthcare) in SSC (Sigma). Probes were PCR amplified from genomic DNA in a reaction mix with ^32^P-dCTP (Perkin Elmer) using primers: (External probe) FRW: ATTTGTGCGCATGAACTGC, REV: GTCGAGCAGTAAGGCTAAAGTAA and (Internal probe) FRW: TGTGCATCTCCACTTTGAGC, REV: CATTAATGCATGGCCAGTTG.

### RNA isolation and Northern blotting

Northern blot was performed using standard protocol. Briefly, RNA was isolated from frozen tissues, tumour pieces and cell pellets using TRIzol reagent (Ambion) and RNEasy columns (Qiagen). 10 μg of total RNA was resolved on agarose/formamide gel at 20mV for 6hrs; transferred to Amersham Hybond N^+^ membrane (GE Healthcare) in 20xSSC (Sigma), baked for 1.5hrs at 80°C and UV–crosslinked. Probe was PCR amplified from cDNA template in reaction mix with ^32^P-dCTP (Perkin Elmer) using primers: Internal-FRW: 5’ CAGACGAGGGTTCAGAGAGG, Internal-REV: 5’ TTCTTCCAGAAAGTATGTTTGAACG; and 3’ UTR- FRW: 5’CAGACGAGGGTTCAGAGAGG, 3’UTR-REV: 5’ACATCGAGTTCAGAAACGTATGG. Prehybridization (1hr) and hybridization (o/n) with the probe was done at 65°C in Church buffer. Membrane was washed consecutively in 2xSSC, 2xSSC 0.1%SDS, 0.5xSSC 0.1%SDS buffers, exposed on phosphor screen, and image was acquired on phosphorimager.

### Protein isolation and Western blotting

Western blot was performed using standard protocol. Briefly, cells were trypsinized, pelleted, washed in ice-cold PBS, lysed in RIPA buffer on ice, briefly sonicated and cleared by centrifugation. Protein was resolved on 3–8% gradient Tris-Acetate precast NuPAGE gels (Invitrogen) and transferred onto 0.45 μm Immobilon-P PVDF membrane (Merck Millipore) in 2x Tris-Glycine transfer buffer supplemented with 0.01% SDS for 16hrs at 100mA at 4°C. Membrane was blocked for 4hrs in 5% skimmed milk in TBST at 4°C. Rabbit anti-CHD9 antibody (Bethyl Laboratories, A301-226A) or mouse anti-Vinculin (V9131, Sigma) was incubated in blocking buffer at 1:1000 dilution o/n at 4°C. After three washes with TBST, antibody was incubated with 1:10.000 dilution of secondary goat anti-rabbit antibody coupled to horseradish peroxidase for 1hr at ambient temperature, followed by three washes in TBST. Immuno-complexes were visualised by incubation in Pierce enhanced chemiluminescent (ECL) blotting substrate (Thermofisher).

### Immunofluorescence and immunohistochemistry

MEFs were plated in 8-well glass slide chamber system (Labtek), washed in PBS, fixed in PBS/ 3.7% formaldehyde (Merck), permeabilised with PBS/ 0.1% Triton X-100 (Sigma) and blocked in 10%FBS/PBS. The rabbit anti-CHD9 antibody (Bethyl Laboratories, A301-226A) at dilution 1:1000 was incubated o/n at 4°C, followed by goat anti-rabbit secondary antibody conjugated to Alexa Fluor 488 (1:500); chromatin was counterstained with DAPI (Thermo Scientific), and mounted with Vectashield (Bioconnect). Images were acquired on Leica SP5 Scanning Confocal Microscope.

Tissues were fixed in either 4% formaline or acidified formaldehyde (ethanol/acetic acid/formaldehyde/saline at 40:5:10:45 v/v). Haematoxylin and eosin (H&E) stainings and immunohistochemical procedures were performed according to standard procedures. Sections were prepared at 2 μm thickness from the paraffin blocks and stained with hematoxylin and eosin (HE) according to standard procedures. For immunohistochemistry (IHC), 4 μm -thick sections were made. Primary antibodies were obtained from the following sources: B220 (NKI, 1:4000), Pax5 (Genetex, GTX63400, 1:250), CD3 (Neomarkers, RM-9107-S, 1:600), Myeloperoxidase (DakoCytomation, A0398, 1:600), F4/80 (Serotec, MCA497A, 1:200). Antibody staining was revealed using either diaminobenzidine or 3-amino-9-ethylcarbazole chromogen. Images were captured using a Zeiss Axioskop2 Plus microscope (Carl Zeiss equipped with a Zeiss AxioCam HRc digital camera) and processed using AxioVision 4 software (Carl Zeiss Vision).

### Derivation of MEFs and ES cells

Mouse embryonic fibroblasts (MEFs) were isolated from the *Chd9*^*+/-*^ x *Chd9*^*+/-*^ cross at day 13.5 post coitum and cultured using standard methods. Briefly, heads and organs of embryonic day 13.5 embryos were removed, and the remaining fetal tissue was minced, rinsed in PBS, and incubated for 1 hr in 0.5 ml of 0.1% trypsin/EDTA solution at 37°C. Subsequently, the cell aggregates were dissociated in DMEM supplemented with 10% FBS (GIBCO) and 1% penicillin-streptomycin (GIBCO). After removal of large clumps, the cells were plated in a 175-cm^2^ flask. After 2–3 days, confluent cultures were frozen down and considered as being passage 1.

Embryonic stem cells (ESC) were derived and cultured from the *Chd9*^*+/-*^ x *Chd9*^*+/-*^ cross as described before [48]. ESCs were cultured on 0.1% gelatin-coated plates in N2B27 medium (DMEM/F12 from Gibco, Neurobasal medium, N2 supplement and B27 supplement from Invitrogen) with LIF (Chemicon) and 2i, i.e. 1 μM PD0325901 (Axon Medchem) and 3 μM CHIR99021 (Axon MedChem). ESCs were split between 1:4 and 1:8 every 2–3 days and dissociated with Accutase (Sigma). Cells were cultured at 37°C in 5% CO2. For all the experiments early passage MEFs and ESCs were used.

### Cell culture conditions

Cells were grown at 37°C, 5% CO_2_, either in DMEM (GIBCO) supplemented with 10% FCS, 100 units per ml penicillin, 100 mg/ml streptomycin or in RPMI- 1640 (GIBCO) supplemented with 10% FCS, 100 units per ml penicillin, 100 mg/ml streptomycin. K562 and SNB19 cells were available within The Netherlands Cancer Institute, originally purchased from American Type Culture Collection (ATCC), and confirmed to be mycoplasma free.

SNB19 cells were cultured in DMEM (Invitrogen) and K562 in RPMI-1640 (Invitrogen) media supplemented with 10% FBS and Pen/Strep (Gibco). For doxycycline induction of pLKO-tet-shRNA constructs, cells were grown in same media supplemented with 10% Tet system approved FBS (Clontech) and 100 units per ml penicillin, 100 mg/ml streptomycin. Knockdown was induced by adding 1 μg/ml doxycycline (Sigma). 48 hours upon knockdown cells were pelleted for RNA/protein isolation or plated for proliferation assay. The shRNA sequences used in this study are: shRNA-Luciferase: CGCTGAGTACTTCGAAATGTC, shRNA -CHD9#1: TGCATACCAGCGTACTAATAA, shRNA-CHD9#2: CCGAGCTCTCTTAGCATATTG.

#### Proliferation and transformation assays

For proliferation assay, 25.000 MEFs were seeded in a 12-well plate, fixed in 4% PFA and stained with Crystal Violet (Merck) Ethanol solution for 10 min at RT at indicated time points. Stained cells were dissolved in 10% acetic acid, diluted 1:4 in MQ and absorbance was measured at OD590nm. Experiment was performed twice, with two knockout and wildtype MEFs seeded in triplicate. Values represent average ± SEM, two-way ANOVA was used to determine statistical significance.

Proliferation assay of human SNB19 glioblastoma cells with inducible pLKO-tet-shRNA-CHD9 was done using the same protocol, with only difference that prior to seeding cells were treated with 1μg/ml doxycycline (Sigma) for 48hrs. Cells were fixed and processed at indicated time points as described above. Doxycycline was kept for duration of the whole experiment.

Proliferation assay of human K562 cells was done as described above, with one major difference that cells were plated in a 6-well in triplicate and cell number was assayed daily using CASY Cell counter without replating. Doxycycline was kept for duration of the whole experiment. Values represent average ± SEM, two-way ANOVA was used to determine statistical significance.

For soft agar growth analysis, MEFs were first infected with LZRS-TBX2-GFP, followed by infection with Myc, RasV12 and E1A or combinations thereof. Oncogene expression following transduction was confirmed on the Western blot. After selection, 50,000 MEFs were resuspended in 0.4% soft agar and plated on a bottom layer of 1% agar per well of a 6-well ultra-low attachment plate (Corning) in triplicate. The number of colonies growing in soft agar after two weeks of culture was quantified using ImageJ software. Experiment was performed twice, using two wildtype and knockout MEFs, data values are represented as average ± SEM, p-value calculated using two-tailed Student’s t-test.

All statistical analyses of cell proliferation and transformation was done using GraphPad Prism8. For all calculations of p-values, significance threshold alpha was set at 5% (p< 0.05).

### FACS analysis of hematopoietic lineages

#### Hematology

Peripheral blood was collected into EDTA-coated microtubes and analyzed by FACS. Blood was depleted from red blood cells by hypotonic lysis and staining was performed with fluorochrome-labelled antibodies specific for B220, CD3, CD11b. Samples were analyzed on the LSR Fortessa cell analyzer (BD).

#### Flow cytometric analyses of hematopoietic subpopulations

Flow cytometric analysis was performed as described previously [49]. BM mononuclear cells (MNCs) were stained for lymphoid and myeloid lineage markers. To analyze and isolate BM LSK, LT-HSC, ST-HSC, MPP, CLP, and CMP subpopulations, MNCs were first stained with Lineage Cell Detection Cocktail-Biotinylated mouse antibody (Macs; Miltenyi Biotec). For FACS analysis, cells were then directly stained with fluorochrome-conjugated antibodies against Sca1 (Pacific blue), c-kit (APC), CD34 (FITC), CD135/Flk2 (PE), CD150 (PECy7), CD16/32 (PerCpCy5,5), and CD127 (PerCpCy5,5). For cell sorting of LSK, LT-HSC, and ST-HSC, depletion of lineage positive cells was performed using Biotin MicroBeads (Macs; Miltenyi Biotec) and magnetic columns (Macs; Miltenyi Biotec) before staining. Samples were analyzed with CyAn ADP Analyzer. Cell sorting was performed with a FACSAria cell analyzer (BD). All FACS data were analyzed using FlowJo Software (Tree Star). For all FACS analysis p-value was calculated using two-tailed Student’s t-test in Microsoft Excel. For all calculations of p-values, significance threshold alpha was set at 5% (p< 0.05).

#### Antibody list

*Blood lymphoid*. CD4-Fitc (BioLegend), CD8-PE-Cy7 (BD), B220–Pacific blue (BD), CD11b-PerCpCy5,5 (BD), CD3-APC (BioLegend); *Blood myeloid and erythroid*: CD3-Fitc (BD), CD19-Fitc (BD), B220-Fitc (BD), Gr1-PE (BD), F4/80-Pacific blue (BioLegend), CD11b-APC (BD);

BM lymphoid. IgD-Fitc (BD), CD25-PE (BioLegend), CD11b-PerCpCy5,5 (BD), IgM-PECy7 (Southern Biotech), B220-Pacific blue (BD), cKit-APC (BD); *BM myeloid*: Gr1-PE (BD), CD11b-PerCpCy5,5 (BD), Ter119-PECy7 (BD), F4/80–Pacific blue (BioLegend), CD19-APC (BioLegend); *BM megakaryocyte*: CD3-Fitc (BD), CD41-PE (BioLegend), CD11b-PerCpCy5,5 (BD), B220–Pacific blue (BD), CD19-APC (BioLegend); HSPC and CLP: Strep-APC/CY7 (Southern Biotech), cKit-APC (BD), SCA1-PacBlue (BioLegend), CD34-FITC (eBioscience), CD135-PE (BioLegend), CD150-PECy7 (BioLegend), CD127-PerCpCy5,5 (BioLegend); *Hematopoietic progenitors*: Strep-APC/CY7 (Southern Biotech), cKit-APC (BD), SCA1-PacBlue (BioLegend), CD34-FITC (eBioscience), CD16/32-PerCpCy5,5 (eBioscience), CD127-PE (eBioscience).

### RNA-seq analysis

Tissues from individual E15 embryos (brain, liver), derived cells (MEF, ESC), *EμMyc* lymphomas or human cancer cell lines (SNB19 and K562) were isolated and total RNA was extracted. Samples were prepared using TruSeq protocol, and standard sample preparation protocols and RNA-seq was performed on a Hiseq2000 machine (Illumina) at the NKI Genomics Core Facility.

#### Bioinformatic analysis

Sequencing data were aligned to mm9 (MEFs, ESCs), mm10 (E15 brain and liver; *EμMyc* lymphomas), hg37 (K562 and SNB19 cells) using TopHat (v2.1.0 using bowtie 1.1.0). and number of reads per gene were measured with HTSeq count (v0.5.3). Statistical analysis of the differential expression of genes were performed using DESeq2 [50]. Results were considered statistically significant at an adjusted p < 0.05. Normalized expression values were obtained by correcting for differences in sequencing depth between samples using DESeqs median-of-ratios approach. The signature gene set for Polycomb repressive complexes (PRC1 and PRC2) and Chromodomain helicase DNA-binding (CHD) proteins were obtained from previously published studies [51][12].

For gene set enrichment analysis using Generally Applicable Gene-set enrichment for pathway analysis (GAGE) was used [52]. The x-axis represents enrichment score and the size of the dots indicates the corrected p values (p.adj). An enrichment score indicates over-representation (positive) or under-representation (negative) of the DEGs in a given pathway.

## Supporting information

S1 Fig Generation of the *Chd9* knockout allele by sequential targeting and Cre-mediated deletion. (A) Representation of the targeting strategy whereby recombineering–generated cassettes with loxP sites were introduced sequentially by homologous recombination at the 5’ and 3’ region of the *Chd9* allele. *Cre* recombinase excision removed 5’ NEO cassette to allow subsequent 3’ targeting with FRT–loxP-NEO cassette, and Flp–mediated removal in-vivo. Heterozygous mice were generated by breading with universal β-actin *Cre* recombinase strain, resulting in the excision of the intervening 117kb genomic DNA and removal of the coding potential of the locus (exon 3—exon 39). Southern blot hybridization in Fig 1. detects DNA fragment of 6.7kb in the wild-type, and 7.4kb in *Chd9*–null allele following Cre–mediated deletion. loxP sites (red triangle), FRT sites (blue triangle), Neomycin selection cassette (grey rectangle labelled *Neo*), *Cre* recombinase (Cre), *Flp* recombinase (Flp). HindIII restriction sites(red), genomic location of the PCR probe used for Southern blot (green rectangle). (B) Integrative Genomics Viewer (IGV) snapshot of the region surrounding the loxP site flanking exon3 (left) and region surrounding the 3’ UTR loxP site (right). The loxP sites are 117kb apart. Active transcription within the locus that gives rise to the 4.2kb mRNA species is represented by RNA–seq reads from wildtype (red) and knockout (blue) MEFs.
(PDF)

S2 Fig Immunoflourescence staining of the CHD9 protein in primary murine embryonic fibroblasts (MEFs). Endogenous CHD9 protein is localized in distinct nuclear foci that are absent in the knockout MEFs. Background cytoplasmic signal remains visible in the knockouts. Dashed white lines indicate nuclei boundaries. (Green signal CHD9, Rabbit anti-CHD9 Bethyl-labs, Alexa488), chromatin (DAPI, Blue signal). Scale bar 10μm (white line).
(PDF)

S3 Fig Gene expression analysis of embryonic day 15 (E15) liver and brain of *Chd9*^*-/-*^ animals. (A) Liver, (B) Brain (anticlockwise): Volcano plots represent differentially expressed genes (DEGs) between *Chd9*^*-/-*^ and *Chd9*^*+/+*^ liver (A), brain (B). The DEGs with p.adj <0.05 and log2 (fold change) > ±1 are shown, *Chd9* gene is highlighted in orange. Heatmap of statistically significant DEGs in *Chd9*^*-/-*^ compared to control *Chd9*^*+/+*^ organs. The color scale is based on normalized read values. Scatter-plot expression profiles of each gene in the Polycomb repressive complex 1 (PRC1) and 2 (PRC2) and CHD family shown based on the log2 fold change over the average expression strength. Genes highlighted in red are found to be significant (p.adj < 0.05).
(PDF)

S4 Fig Gene expression analysis of murine embryonic fibroblasts (MEF) and embryonic stem cells (ESC) from *Chd9*^*-/-*^ animals. (A) MEFs, (B) ESCs (anticlockwise): Volcano plots represent differentially expressed genes (DEGs) between *Chd9*^*-/-*^ and *Chd9*^*+/+*^ MEFs (A), ESCs (B). The DEGs with p.adj <0.05 and log2 (fold change) > ±1 are shown, *Chd9* gene is highlighted in orange. Heatmap of statistically significant DEGs in *Chd9*^*-/-*^ compared to control *Chd9*^*+/+*^ organs. The color scale is based on normalized read values. Scatter-plot expression profiles of each gene in the Polycomb repressive complex 1 (PRC1) and 2 (PRC2) and CHD family shown based on the log2 fold change over the average expression strength. Genes highlighted in red are found to be significant (p.adj < 0.05).
(PDF)

S5 Fig Pseudogene Gm13237 does not affect Hmgb1 and Hmgb2 protein levels in *Chd9* knockout MEFs. (A) Gm13237 is a processed pseudogene (635bp), located on mouse Chromosome 4: 145,632,952–145,633,586, forward strain (mm9). It originates through genomic integration of reverse–transcribed parental gene mRNA, and bears homology to both human HMGB1 and HMGB2. Upper panel represents snapshot of Integrative Genome Viewer (IGV) RNA–seq reads of *Chd9* knockout (red) and wildtype (blue) MEFs. Lower panel represents genomic location of Gm13237(ENSMUST00000122151) in the UCSC Genome Browser (mm9). (B) Western blot analysis (15% SDS–PAGE) of Hmgb1 and Hmgb2 proteins in *Chd9* knockout and wildtype MEFs shows no difference in protein abundance and isoform expression. Gapdh is used as a loading control.
(PDF)

S6 Fig Flow cytometric characterization of the *Chd9* knockout spleen, thymus and peripheral blood major cell populations. (A) Flow cytometric analysis of the *Chd9* wild-type (WT) and knockout (KO) thymi: Left panel, frequency of the CD4^+^CD8^+^ double positive effector memory T-cells and naive CD4^+^ and CD8^+^ single positive cells. Middle panel, frequency of the immature double–negative (DN) cell subset categories, based on their expression of CD44 and CD25 surface markers: CD44^+^CD25^–^ (DNI), CD44^+^CD25^+^ (DNII), CD44^–^CD25^+^ (DN III), and CD44^–^CD25^–^ (DNIV). Right panel, total count of cells in the wild–type and knockout thymi. Bellow, frequency of the CD4^+^ (left) and CD8^+^ T-cells (right) expressing TCR-β (T-cell beta) receptor.(B) Flow cytometric analysis of the Chd9 WT and KO spleens: Far left panel, frequency of the CD3^+^ pro-thymocytes. Left panel, frequency of the IgM/IgD double positive naive mature B-cells. Right, frequency of the CD4 and CD8 double negative, single positive and double positive T-cells. Absolute number of cells in spleens of *Chd9* WT and KO animals. (C) Flow cytometric analysis of the peripheral blood populations in the *Chd9* WT and KO animals: Left, frequency of the lymphoid and myeloid cells, B- and T-cells (middle) and white blood cells (eosinophils, monocytes and neutrophils). Data plotted as frequency of hematopoietic cells, mean ±SD, p-value calculated using two-tailed Student’s t–test.
(PDF)

S7 Fig Characterization of the *EμMyc* organs and lymphomas. (A) Autoradiogram of Northern blot analysis of *Chd9* mRNA levels in tissues derived from wildtype and *EμMyc;Chd9*^*-/-*^ mice using probe spanning 3’UTR. Active transcription within the knockout *Chd9* locus is revealed (labelled with *). Detected mRNA from the *Chd9*^*-/-*^ allele migrates at the size of ~4.2kb, corresponding to the size of the remaining exons within the locus (ex1-3, ex39-40). *Chd9* has two alternatively spliced mRNA isoforms (labelled: a, b). Agarose gel shows Ethidium Bromide–stained rRNA as loading control. (B) Microphotographs of a representative B-cell lymphoma (lymphoblastic lymphoma) from the *EμMyc;Chd9*^*-/-*^ animal, showing stainings of hematoxylin-eosin (H&E), immunohistochemistry (IHC) of the B-cell markers B220 and PAX5, and IHC of general T-cell marker CD3 and leukocyte marker myeloperoxidase. All scale bars: 20mm, except for the upper right one which is 10mm. (C) Volcano plots show differentially expressed genes (DEGs) in *EμMyc;Chd9*^*+/-*^ (left) and *EμMyc;Chd9*^*-/-*^ (right) compared to control *EμMyc;Chd9*^*+/+*^ lymphomas (p.adj <0.05 and log2 (fold change) > ±1). Upregulated genes are highlighted in red, downregulated in blue. DEGs common for *EμMyc;Chd9*^*+/-*^ and *EμMyc;Chd9*^*-/-*^ samples are marked as black dots. *Chd9* is highlighted in orange.
(PDF)

S8 Fig Expression of CHD family members in different murine E15 organs, derived cells and lymphomas and human cancer cell lines. (A, B, C, D, E) Expression profile of each gene is based on the log2 normalized read counts for all samples. *Chd9* is expressed at high levels in all the samples analyzed. In *Chd9* heterozygous and knockout samples, there is a marked reduction in *Chd9* expression. Other family members display variable levels of expression between samples. *Chd4* shows the highest expression in all the samples, whereas *Chd5* is expressed at low levels in MEFs, ESC, E15 liver, and barely detectable in lymphomas. (F, G) In human K562 and SNB19 cancer cell lines *CHD9* is expressed at high levels. Only CHD family members with detectable expression are annotated on the Scatter-plot.
(PDF)

S9 Fig *Chd9* is not required for proliferation or transformation of MEFs. (A) Proliferation of MEFs assayed by crystal violet staining. Left panel shows representative example of a single well stained with crystal violet at indicated time points. Experiment was done twice using two *Chd9* wildtype (WT) and two knockout (KO) clones seeded in triplicate. Right panel is quantification of the crystal violet staining by measuring absorbance at 590nm. Data indicates relative increase in OD_590nm_ absorbance. Day 0 values are set as 1; data represented as average ± standard error mean (SEM), p-value was calculated using two-way ANOVA (ns = not significant, p-value = 0.6816). (B) Anchorage–independent growth was determined by soft agar colony forming assay. Upper panel shows ImageJ colony number quantification of two independent WT and KO clones. MEFs were infected with LZRS-Tbx2-GFP virus, followed by transduction with single or combinations of indicated oncogenes (RasV12, cMyc, E1a). Experiment using two independent WT and KO clones seeded in triplicate was repeated twice (represented as average ± standard-error mean (SEM), p–value calculated using two-tailed Student’s t–test) (ns = not significant). Lower panel shows representative view of the wells containing soft–agar colonies of the transformed WT and KO MEFs used for ImageJ quantification in the upper panel.
(PDF)

S10 Fig *CHD9* is overexpressed predominantly in cancers of neuronal and hematopoietic origin. Gene expression data of *CHD9* across panel of human cancer cell lines, taken from the Broad Institute’s Cancer Cell Line Encyclopedia.
(PDF)

S11 Fig CHD9 depletion deregulates gene expression in human SNB19 and K562 cancer cells. (A) K562 chronic myelogenous leukemia and (B) SNB19 glioblastoma: (left) Unsupervised hierarchical clustering of analyzed knockdown cells, each experiment was done in duplicate. (Right) Volcano plots represent significant differentially expressed genes (DEGs) between two shRNA hairpins targeting CHD9 and control hairpin targeting Luciferase. The DEGs with FDR < 0.05 and log2 (fold change) > ±1 are shown, 20 most significant genes are highlighted (down-blue, up-red); CHD9 gene is highlighted in orange. The DEGs are enumerated at the top of each plot. (Bellow) Proliferation curve representing relative growth of knockdown (shRNA#1 and shRNA#2 hairpins against CHD9) and control (Luciferase hairpin) cells. Cells were treated with 1μg/ml doxycycline (DOX) two days before seeding for proliferation assay. Doxycycline was kept throughout duration of the experiment. In case of K562, cell number was measured on cell counter, whereas for SNB19 glioblastoma relative cell growth was determined by crystal-violet assay. Data represented as average ± standard error mean (SEM), p-value was calculated using two-way ANOVA (for K562 cells p-value = 0.0902, for SNB19 cells p-value = 0.1977, ns = not significant). Cartoon illustrates seeding strategy.
(PDF)

S12 Fig GAGE pathway enrichment analysis for DEGs in primary murine cells, embryonic organs, lymphomas and human cancer cells. Each circle in the scatter plot represents a KEGG pathway, name of the pathway is denoted on the Y-axis and the enrichment score the X-axis. The size of the dots corresponds to the -log10(padj) value, indicating statistically significant KEGG categories. An enrichment score indicates over-representation (positive) or under-representation (negative) of the DEGs in a given pathway. KEGG categories mentioned in the main text are highlighted in red (over-represented), blue (under-represented).
(PDF)

S13 Fig Uncropped images of Western blots. The number of the corresponding Figure in the manuscript is indicated below the image (red rectangle).
(PDF)

S1 Table (XLSX)
