# Supplementary figures and images for: Gene expression regulation by the Chromodomain helicase DNA-binding protein 9 (CHD9) chromatin remodeler is dispensable for murine development
Source: PLoS One. 2020 May 26;15(5):e0233394. doi: 10.1371/journal.pone.0233394 (PMC7250415; doi:10.1371/journal.pone.0233394)

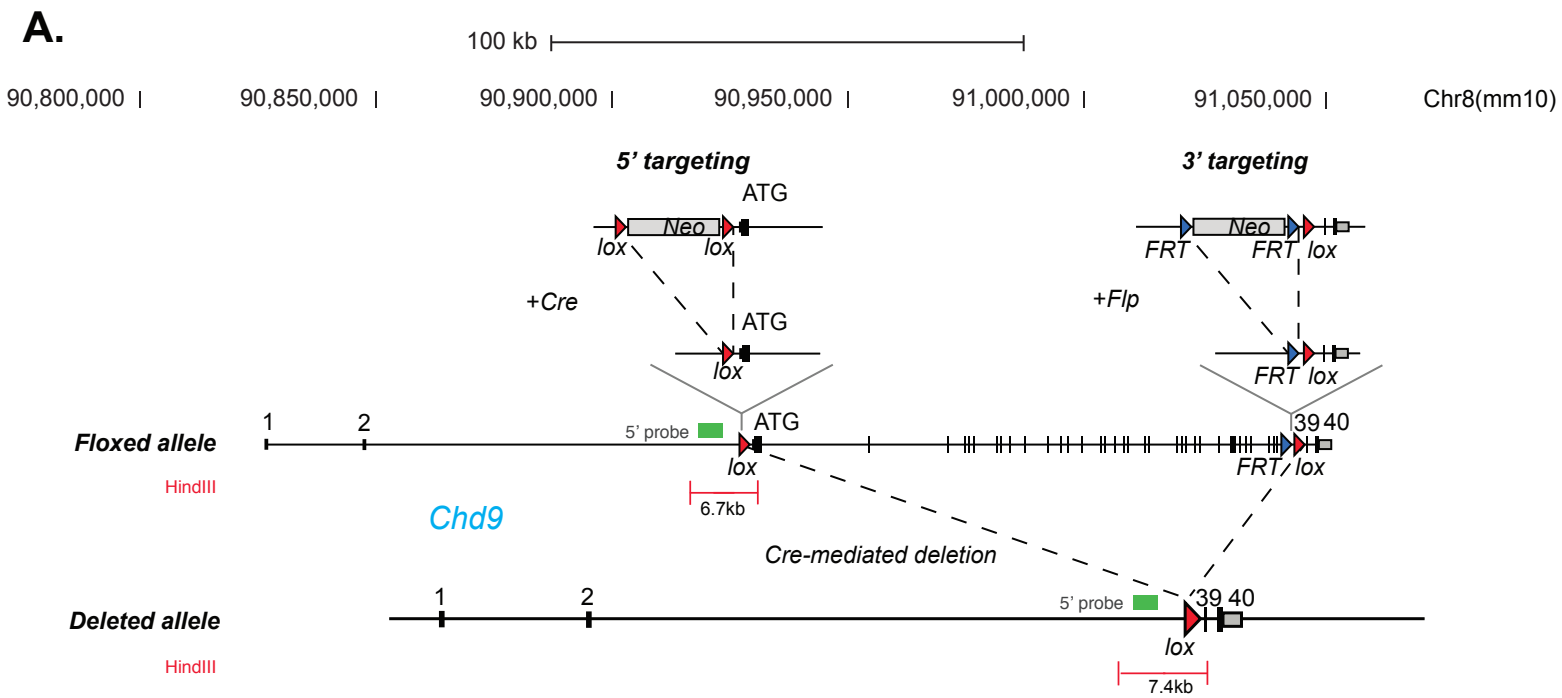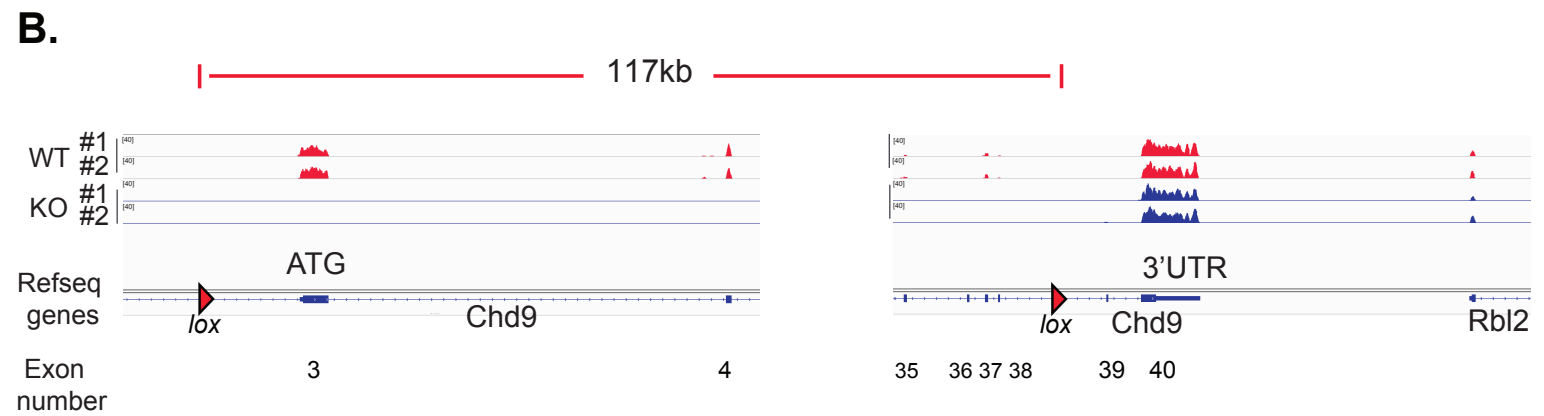

Supplement: S1 Fig — (A) Representation of the targeting strategy whereby recombineering–generated cassettes with loxP sites were introduced sequentially by homologous recombination at the 5’ and 3’ region of the Chd9 allele. Cre recombinase excision removed 5’ NEO cassette to allow subsequent 3’ targeting with FRT–loxP-NEO cassette, and Flp–mediated removal in-vivo. Heterozygous mice were generated by breading with universal β-actin Cre recombinase strain, resulting in the excision of the intervening 117kb genomic DNA and removal of the coding potential of the locus (exon 3—exon 39). Southern blot hybridization in Fig 1. detects DNA fragment of 6.7kb in the wild-type, and 7.4kb in Chd9–null allele following Cre–mediated deletion. loxP sites (red triangle), FRT sites (blue triangle), Neomycin selection cassette (grey rectangle labelled Neo), Cre recombinase (Cre), Flp recombinase (Flp). HindIII restriction sites(red), genomic location of the PCR probe used for Southern blot (green rectangle). (B) Integrative Genomics Viewer (IGV) snapshot of the region surrounding the loxP site flanking exon3 (left) and region surrounding the 3’ UTR loxP site (right). The loxP sites are 117kb apart. Active transcription within the locus that gives rise to the 4.2kb mRNA species is represented by RNA–seq reads from wildtype (red) and knockout (blue) MEFs. (PDF) [file pone.0233394.s001.pdf]

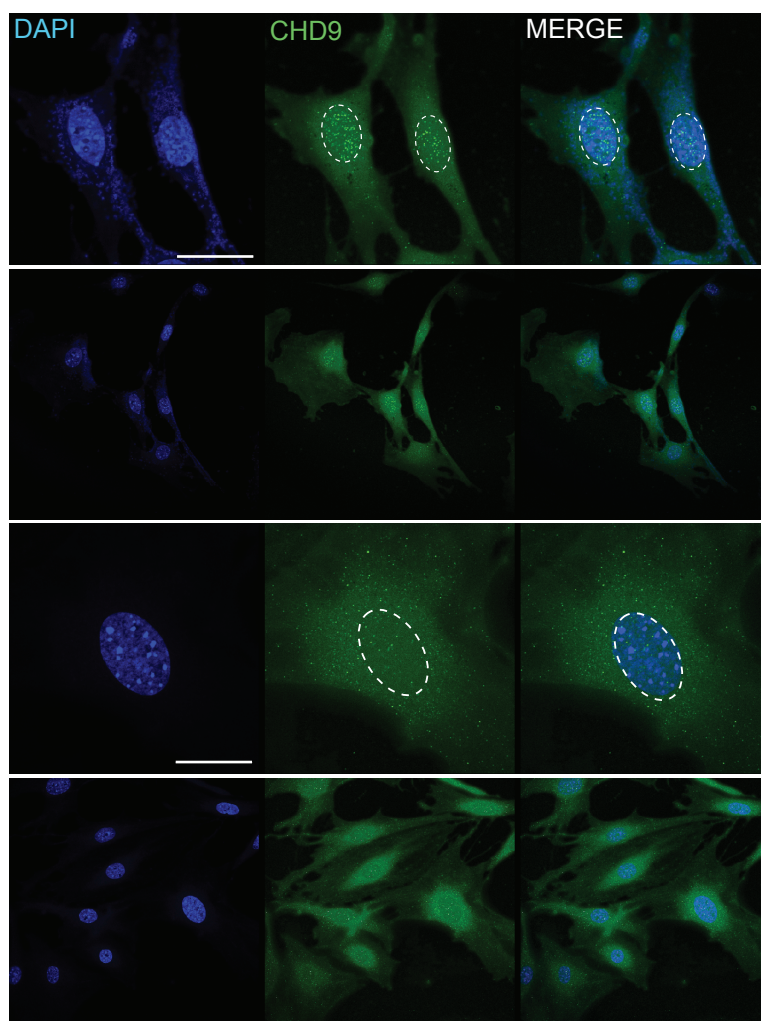

WT MEFs

CHD9 KO MEFs

Supplement: S2 Fig — Endogenous CHD9 protein is localized in distinct nuclear foci that are absent in the knockout MEFs. Background cytoplasmic signal remains visible in the knockouts. Dashed white lines indicate nuclei boundaries. (Green signal CHD9, Rabbit anti-CHD9 Bethyl-labs, Alexa488), chromatin (DAPI, Blue signal). Scale bar 10μm (white line). (PDF) [file pone.0233394.s002.pdf]

A. MEF

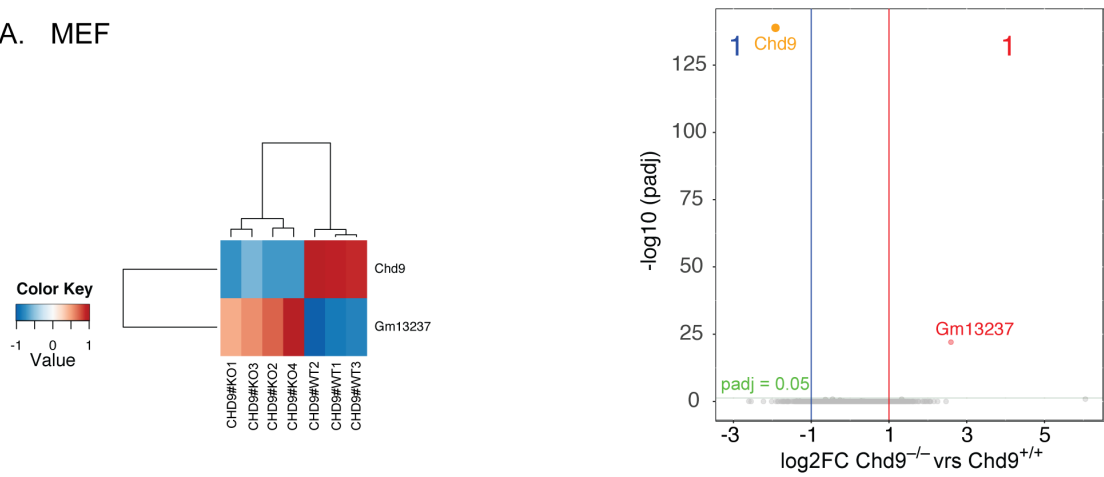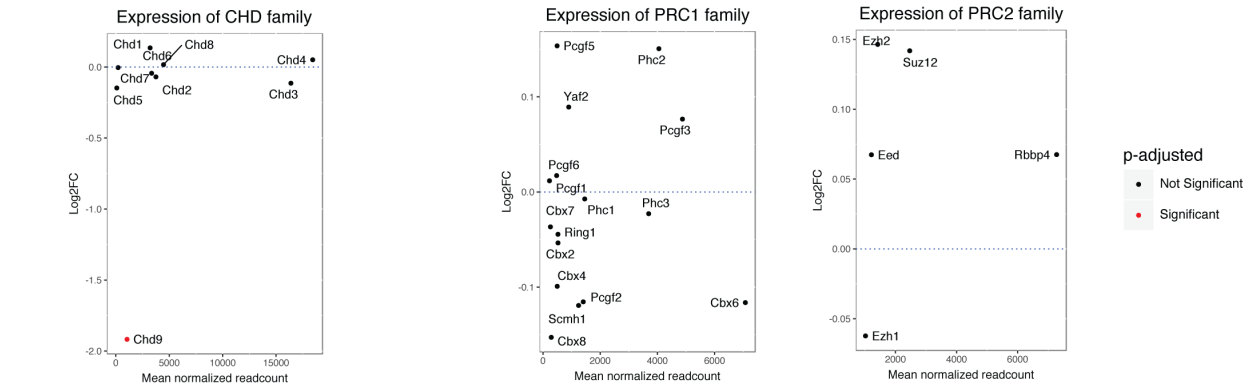

B. ESC

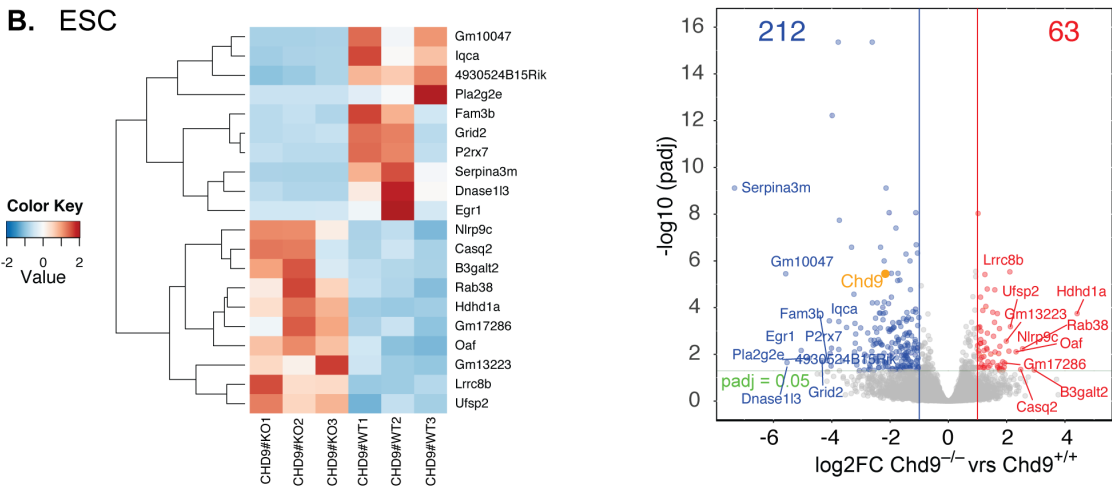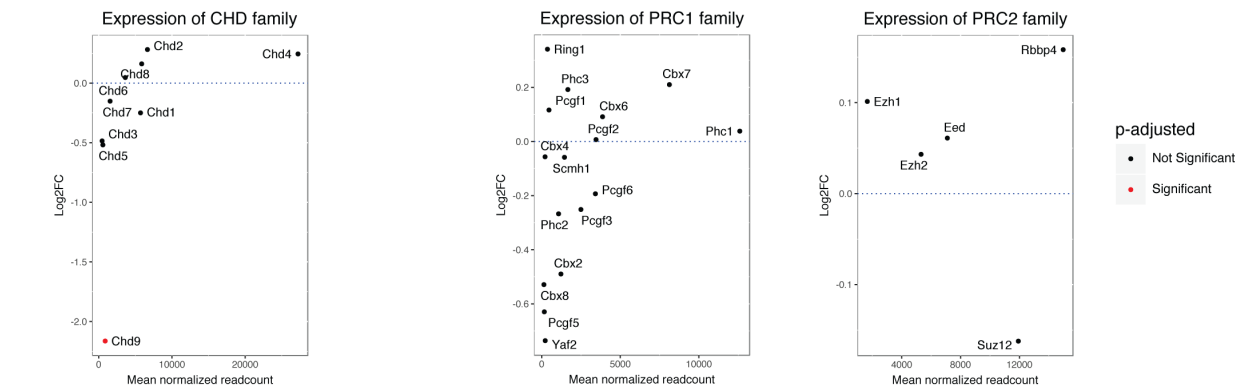

Supplement: S4 Fig — (A) MEFs, (B) ESCs (anticlockwise): Volcano plots represent differentially expressed genes (DEGs) between Chd9-/- and Chd9+/+ MEFs (A), ESCs (B). The DEGs with p.adj <0.05 and log2 (fold change) > ±1 are shown, Chd9 gene is highlighted in orange. Heatmap of statistically significant DEGs in Chd9-/- compared to control Chd9+/+ organs. The color scale is based on normalized read values. Scatter-plot expression profiles of each gene in the Polycomb repressive complex 1 (PRC1) and 2 (PRC2) and CHD family shown based on the log2 fold change over the average expression strength. Genes highlighted in red are found to be significant (p.adj < 0.05). (PDF) [file pone.0233394.s004.pdf]

A.

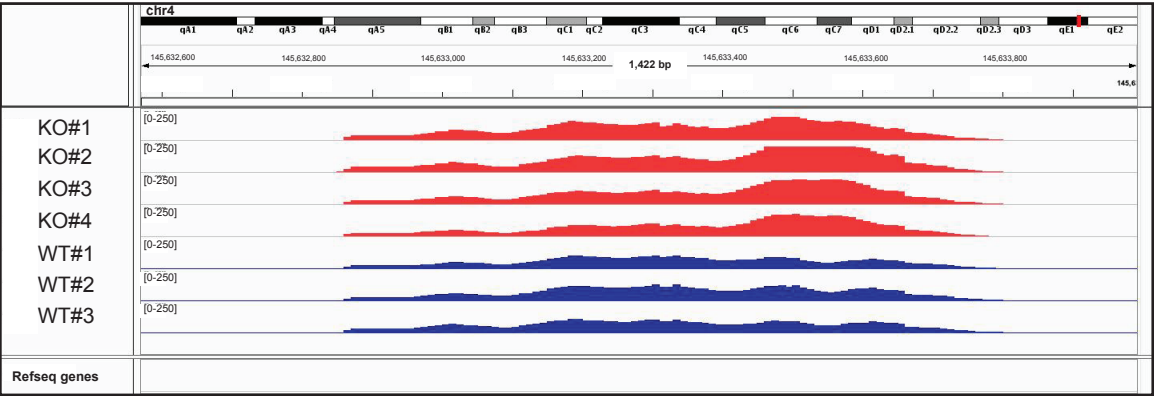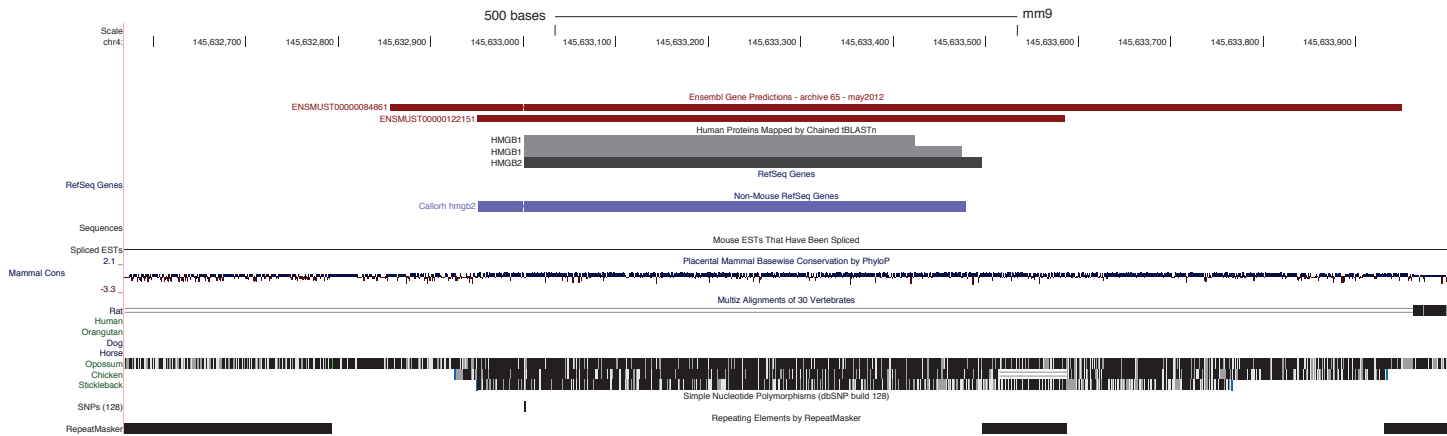

B.

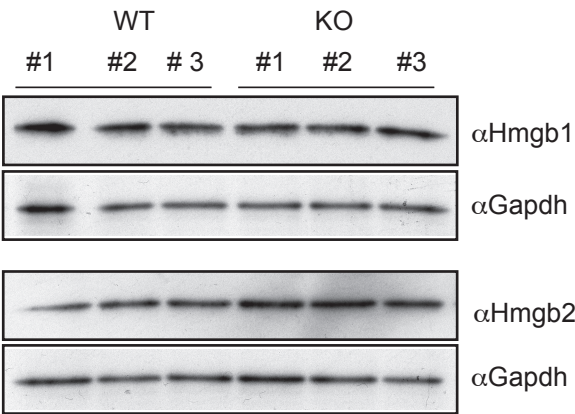

Supplement: S5 Fig — (A) Gm13237 is a processed pseudogene (635bp), located on mouse Chromosome 4: 145,632,952–145,633,586, forward strain (mm9). It originates through genomic integration of reverse–transcribed parental gene mRNA, and bears homology to both human HMGB1 and HMGB2. Upper panel represents snapshot of Integrative Genome Viewer (IGV) RNA–seq reads of Chd9 knockout (red) and wildtype (blue) MEFs. Lower panel represents genomic location of Gm13237(ENSMUST00000122151) in the UCSC Genome Browser (mm9). (B) Western blot analysis (15% SDS–PAGE) of Hmgb1 and Hmgb2 proteins in Chd9 knockout and wildtype MEFs shows no difference in protein abundance and isoform expression. Gapdh is used as a loading control. (PDF) [file pone.0233394.s005.pdf]

## A. Thymus

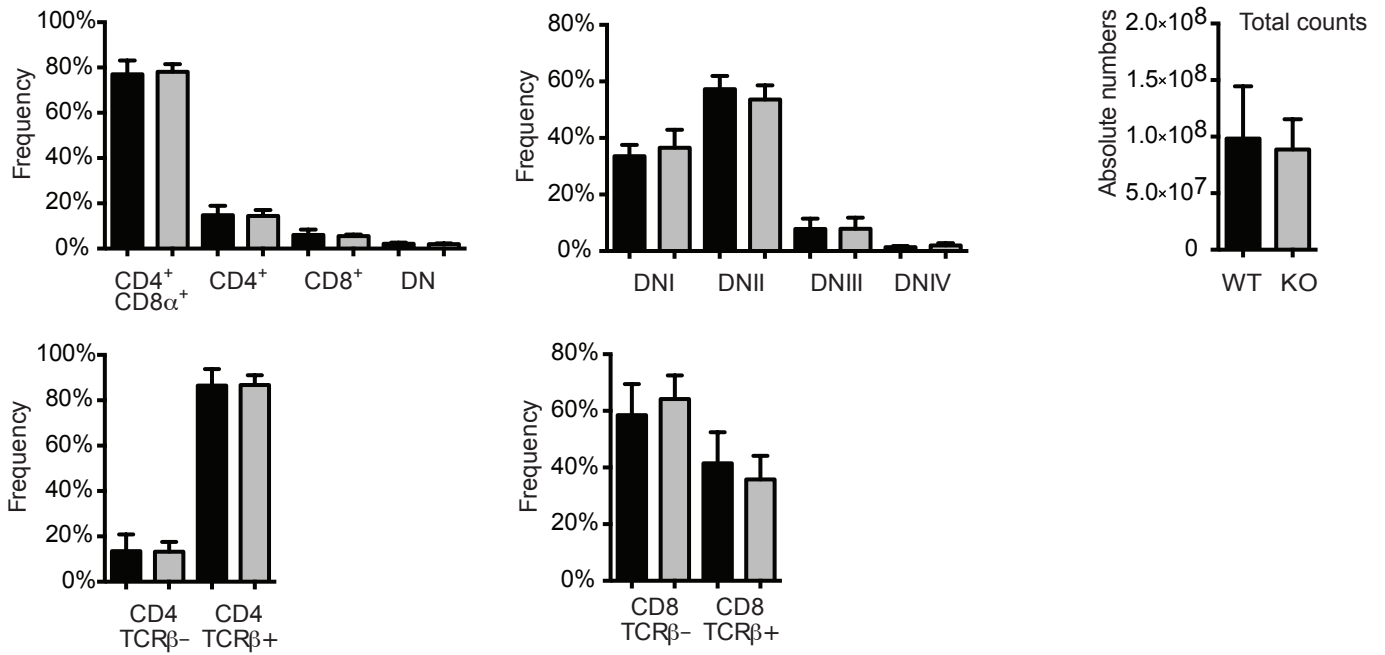

## B. Spleen

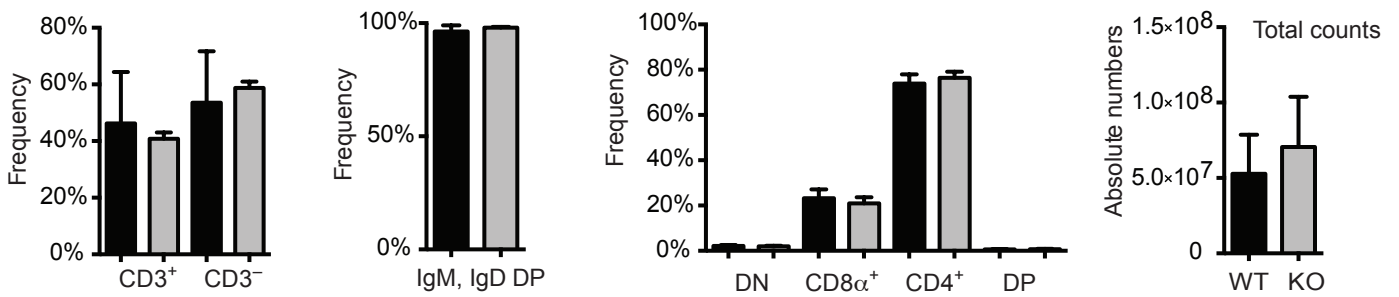

## C. Blood

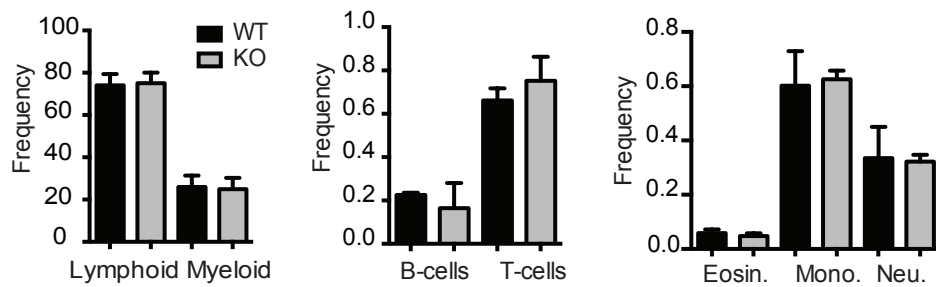

Supplement: S6 Fig — (A) Flow cytometric analysis of the Chd9 wild-type (WT) and knockout (KO) thymi: Left panel, frequency of the CD4+CD8+ double positive effector memory T-cells and naive CD4+ and CD8+ single positive cells. Middle panel, frequency of the immature double–negative (DN) cell subset categories, based on their expression of CD44 and CD25 surface markers: CD44+CD25– (DNI), CD44+CD25+ (DNII), CD44–CD25+ (DN III), and CD44–CD25– (DNIV). Right panel, total count of cells in the wild–type and knockout thymi. Bellow, frequency of the CD4+ (left) and CD8+ T-cells (right) expressing TCR-β (T-cell beta) receptor.(B) Flow cytometric analysis of the Chd9 WT and KO spleens: Far left panel, frequency of the CD3+ pro-thymocytes. Left panel, frequency of the IgM/IgD double positive naive mature B-cells. Right, frequency of the CD4 and CD8 double negative, single positive and double positive T-cells. Absolute number of cells in spleens of Chd9 WT and KO animals. (C) Flow cytometric analysis of the peripheral blood populations in the Chd9 WT and KO animals: Left, frequency of the lymphoid and myeloid cells, B- and T-cells (middle) and white blood cells (eosinophils, monocytes and neutrophils). Data plotted as frequency of hematopoietic cells, mean ±SD, p-value calculated using two-tailed Student’s t–test. (PDF) [file pone.0233394.s006.pdf]

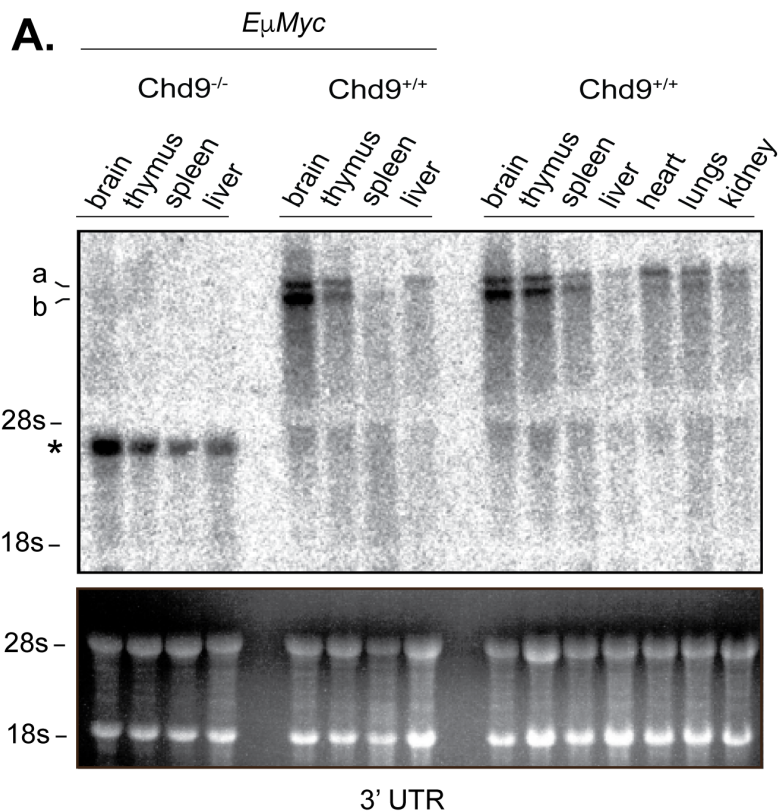

**B.**

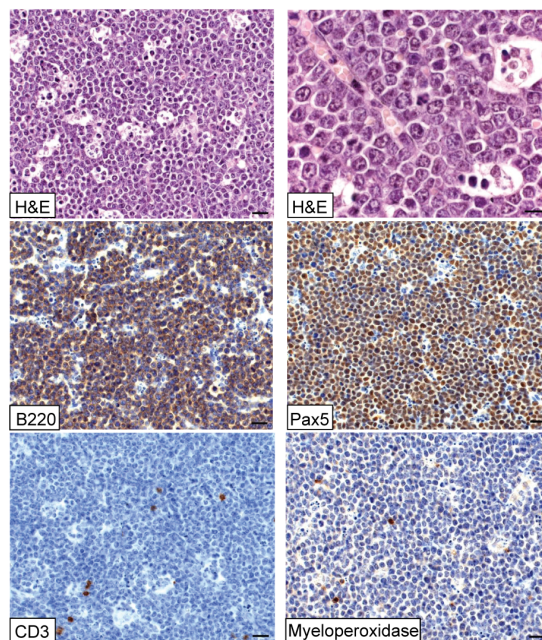

**C.**

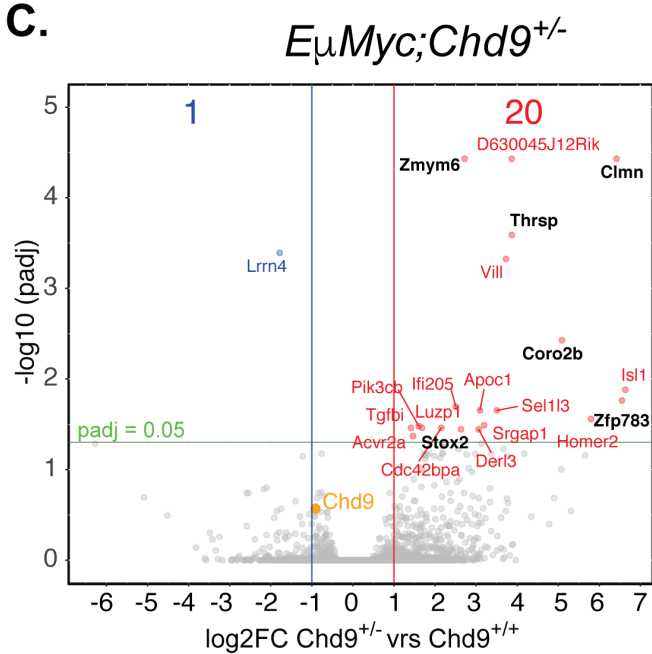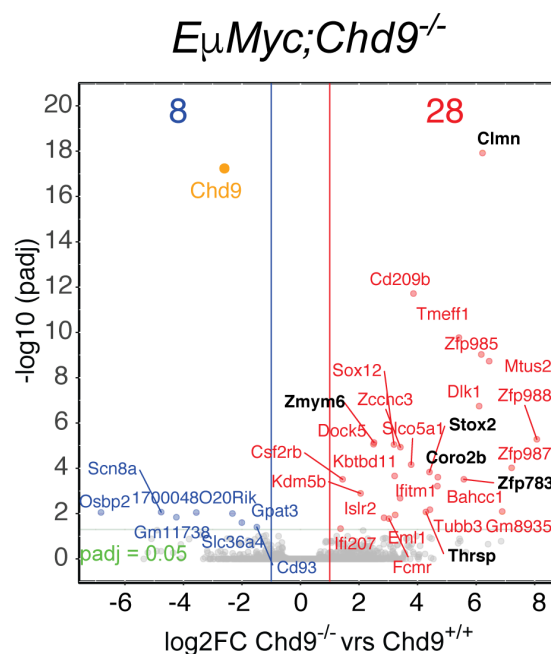

Supplement: S7 Fig — (A) Autoradiogram of Northern blot analysis of Chd9 mRNA levels in tissues derived from wildtype and EμMyc;Chd9-/- mice using probe spanning 3’UTR. Active transcription within the knockout Chd9 locus is revealed (labelled with *). Detected mRNA from the Chd9-/- allele migrates at the size of ~4.2kb, corresponding to the size of the remaining exons within the locus (ex1-3, ex39-40). Chd9 has two alternatively spliced mRNA isoforms (labelled: a, b). Agarose gel shows Ethidium Bromide–stained rRNA as loading control. (B) Microphotographs of a representative B-cell lymphoma (lymphoblastic lymphoma) from the EμMyc;Chd9-/- animal, showing stainings of hematoxylin-eosin (H&E), immunohistochemistry (IHC) of the B-cell markers B220 and PAX5, and IHC of general T-cell marker CD3 and leukocyte marker myeloperoxidase. All scale bars: 20mm, except for the upper right one which is 10mm. (C) Volcano plots show differentially expressed genes (DEGs) in EμMyc;Chd9+/- (left) and EμMyc;Chd9-/- (right) compared to control EμMyc;Chd9+/+ lymphomas (p.adj <0.05 and log2 (fold change) > ±1). Upregulated genes are highlighted in red, downregulated in blue. DEGs common for EμMyc;Chd9+/- and EμMyc;Chd9-/- samples are marked as black dots. Chd9 is highlighted in orange. (PDF) [file pone.0233394.s007.pdf]

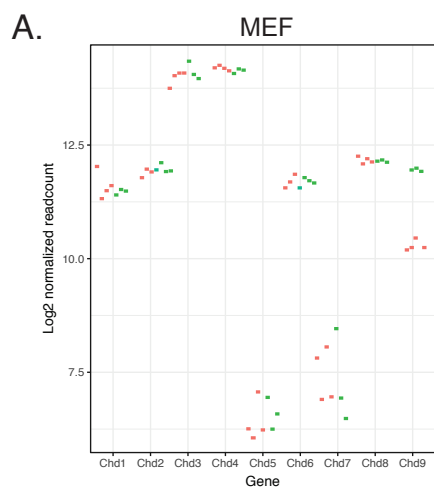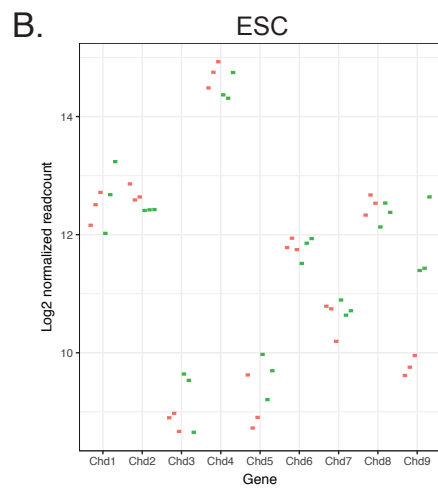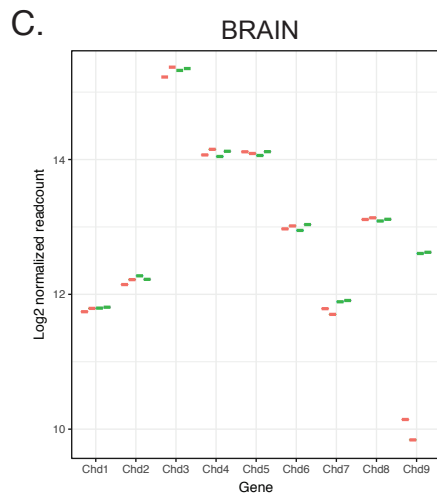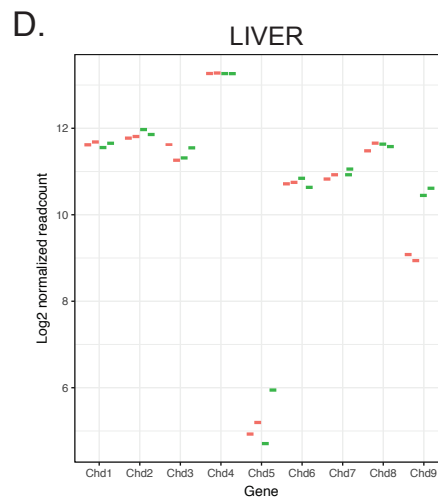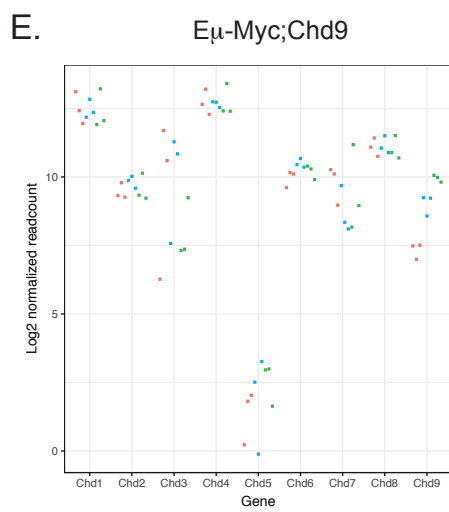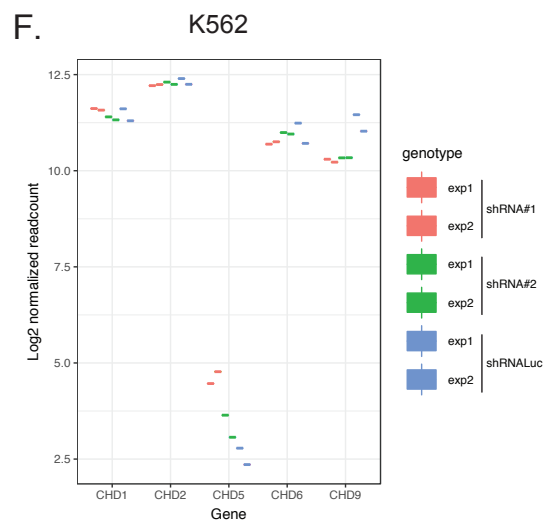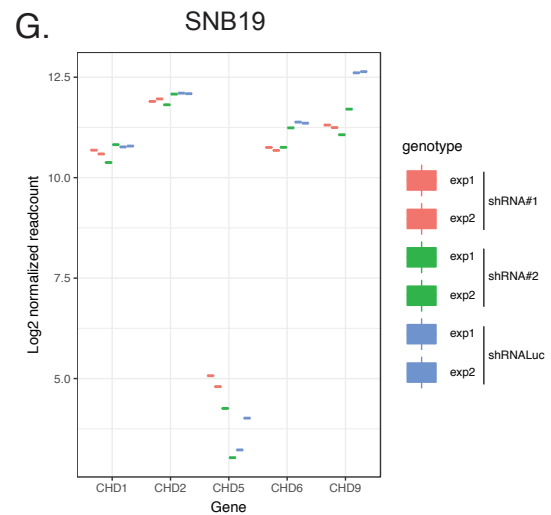

Supplement: S8 Fig — (A, B, C, D, E) Expression profile of each gene is based on the log2 normalized read counts for all samples. Chd9 is expressed at high levels in all the samples analyzed. In Chd9 heterozygous and knockout samples, there is a marked reduction in Chd9 expression. Other family members display variable levels of expression between samples. Chd4 shows the highest expression in all the samples, whereas Chd5 is expressed at low levels in MEFs, ESC, E15 liver, and barely detectable in lymphomas. (F, G) In human K562 and SNB19 cancer cell lines CHD9 is expressed at high levels. Only CHD family members with detectable expression are annotated on the Scatter-plot. (PDF) [file pone.0233394.s008.pdf]

A.

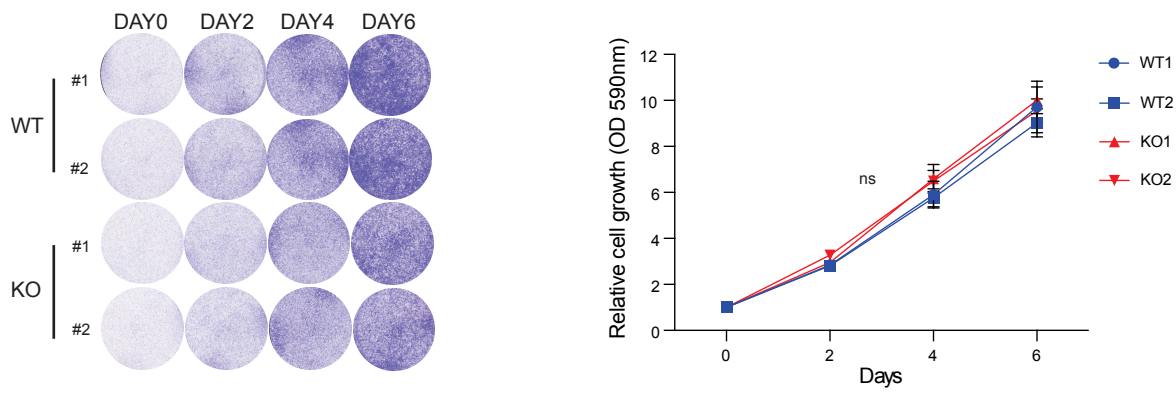

B.

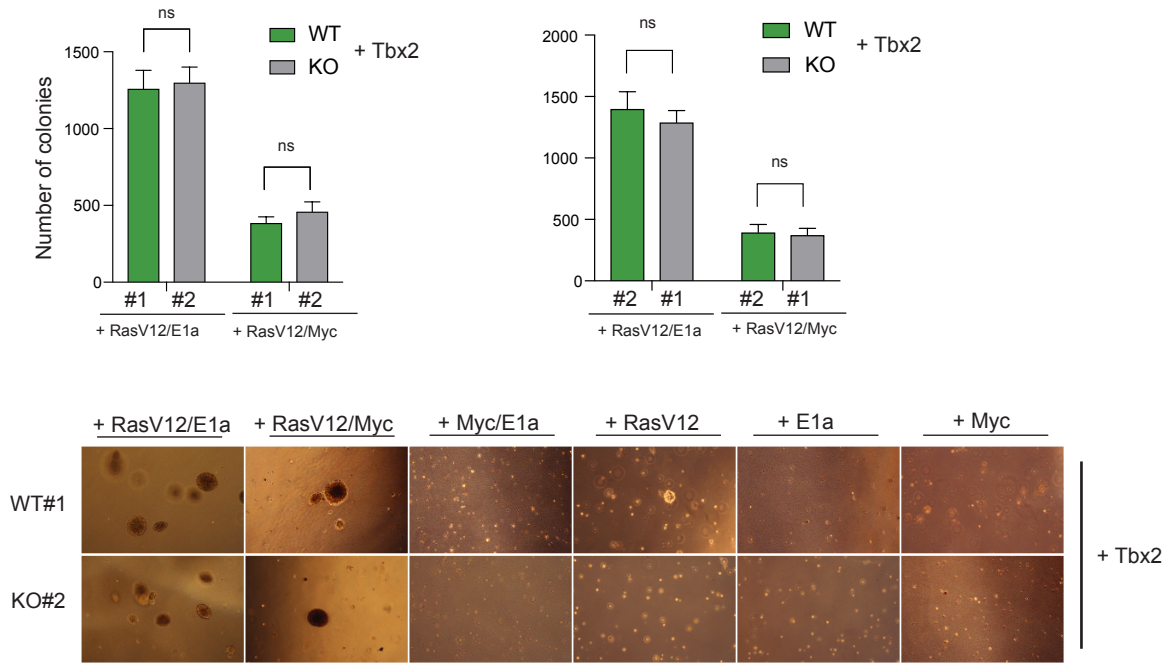

Supplement: S9 Fig — (A) Proliferation of MEFs assayed by crystal violet staining. Left panel shows representative example of a single well stained with crystal violet at indicated time points. Experiment was done twice using two Chd9 wildtype (WT) and two knockout (KO) clones seeded in triplicate. Right panel is quantification of the crystal violet staining by measuring absorbance at 590nm. Data indicates relative increase in OD590nm absorbance. Day 0 values are set as 1; data represented as average ± standard error mean (SEM), p-value was calculated using two-way ANOVA (ns = not significant, p-value = 0.6816). (B) Anchorage–independent growth was determined by soft agar colony forming assay. Upper panel shows ImageJ colony number quantification of two independent WT and KO clones. MEFs were infected with LZRS-Tbx2-GFP virus, followed by transduction with single or combinations of indicated oncogenes (RasV12, cMyc, E1a). Experiment using two independent WT and KO clones seeded in triplicate was repeated twice (represented as average ± standard-error mean (SEM), p–value calculated using two-tailed Student’s t–test) (ns = not significant). Lower panel shows representative view of the wells containing soft–agar colonies of the transformed WT and KO MEFs used for ImageJ quantification in the upper panel. (PDF) [file pone.0233394.s009.pdf]

mRNA expression (Affy): CHD9

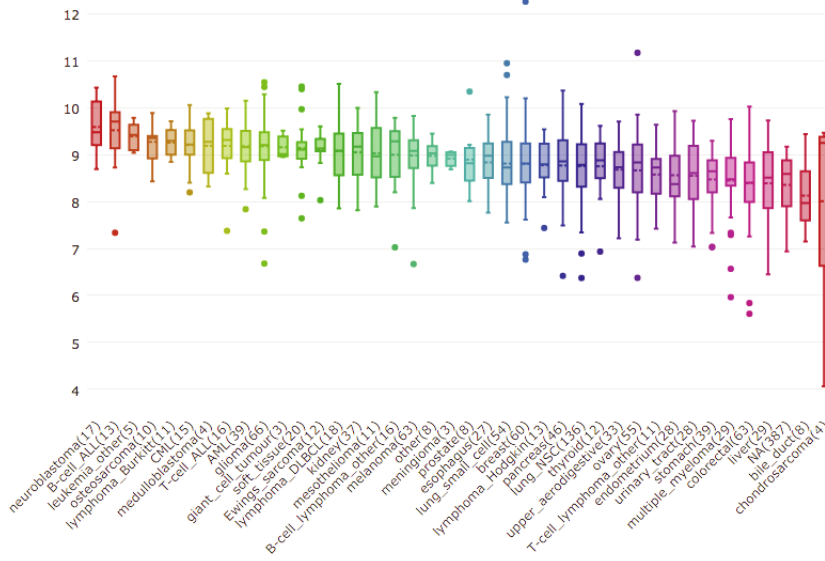

mRNA expression (RNAseq): CHD9

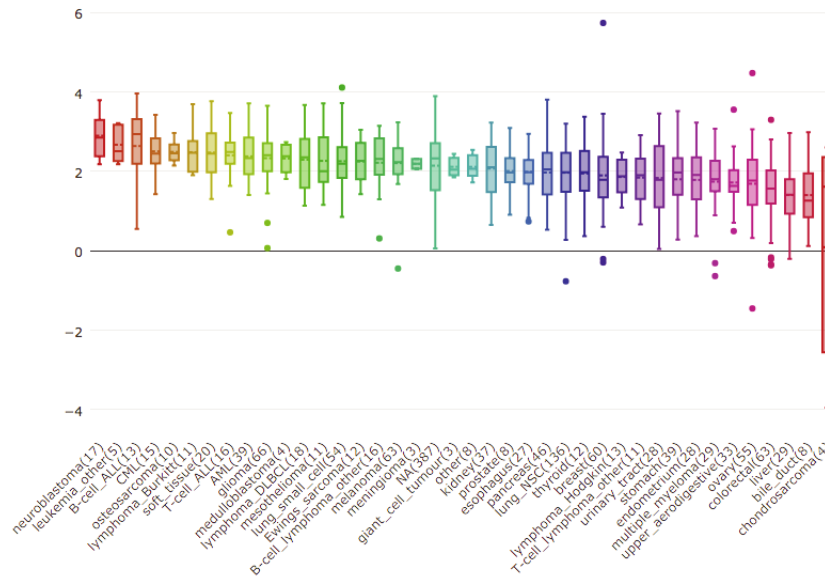

Supplement: S10 Fig — Gene expression data of CHD9 across panel of human cancer cell lines, taken from the Broad Institute’s Cancer Cell Line Encyclopedia. (PDF) [file pone.0233394.s010.pdf]

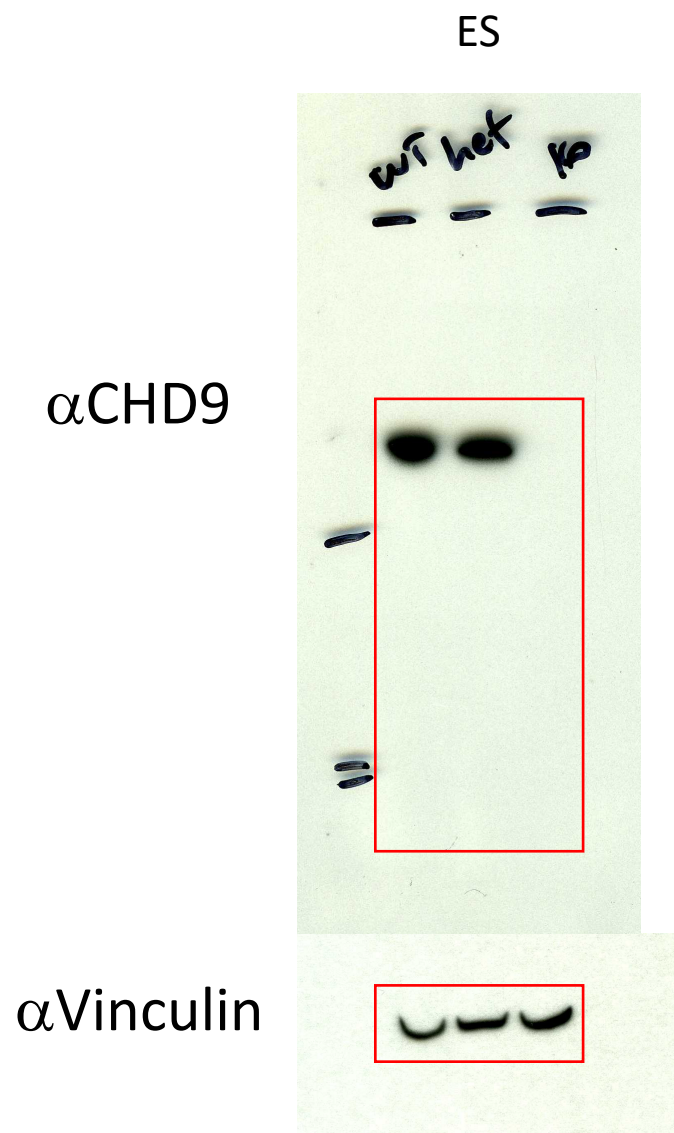

Fig 1C

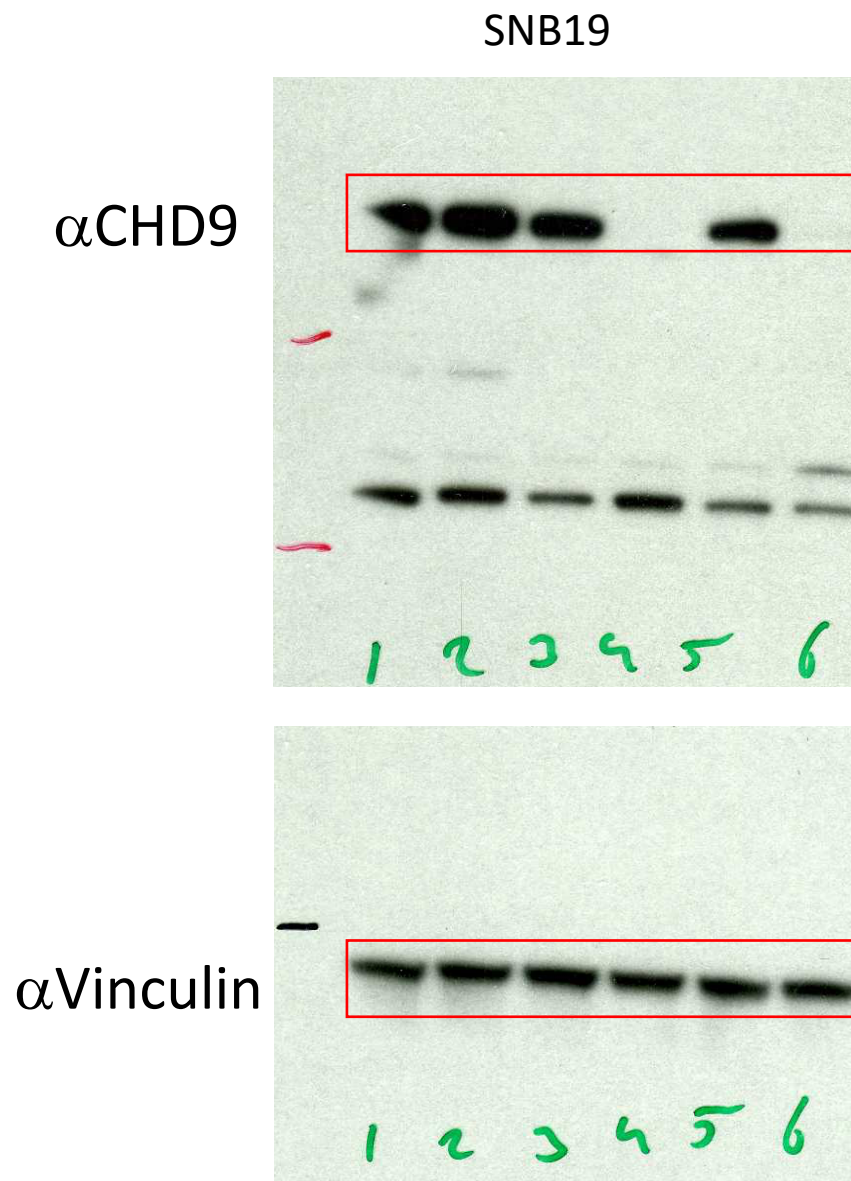

Fig 5A

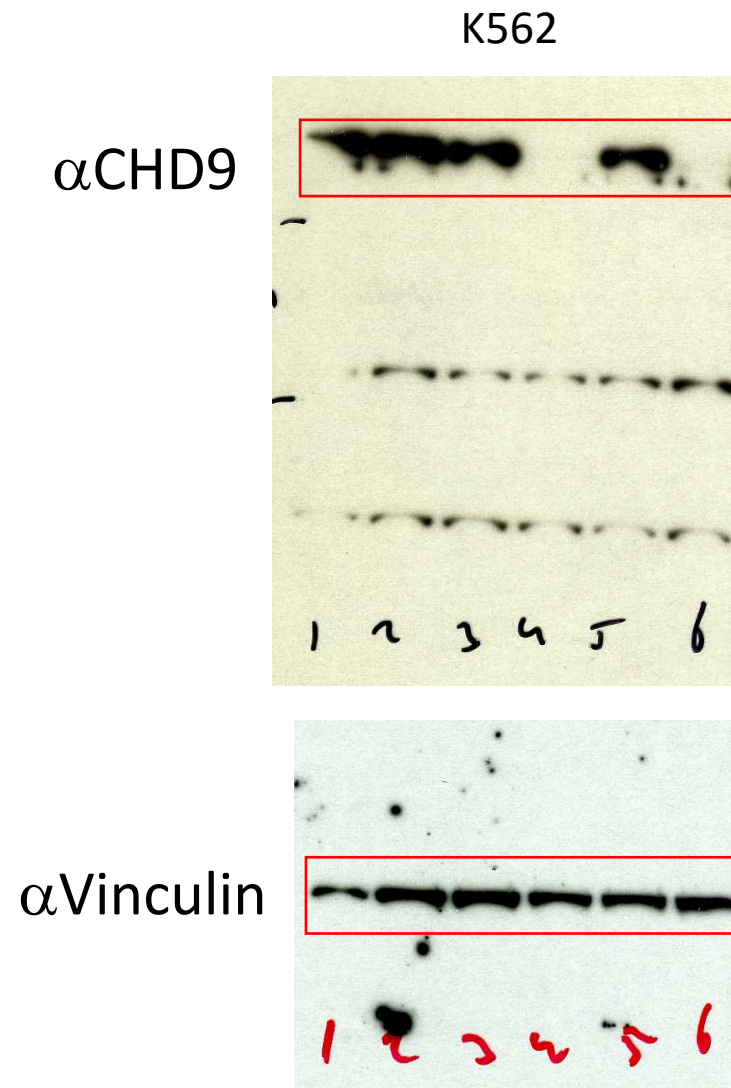

Fig 5B

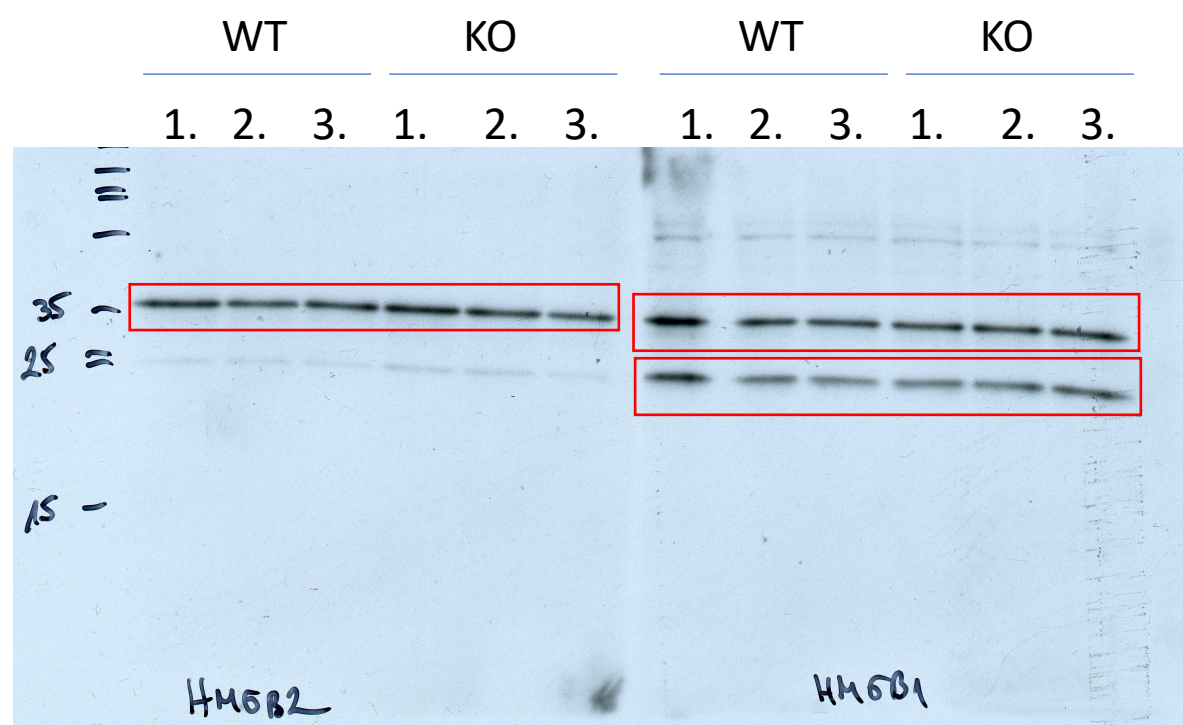

←  $\alpha$ GAPDH (35kDa)

←  $\alpha$ HMGB1 (25kDa)

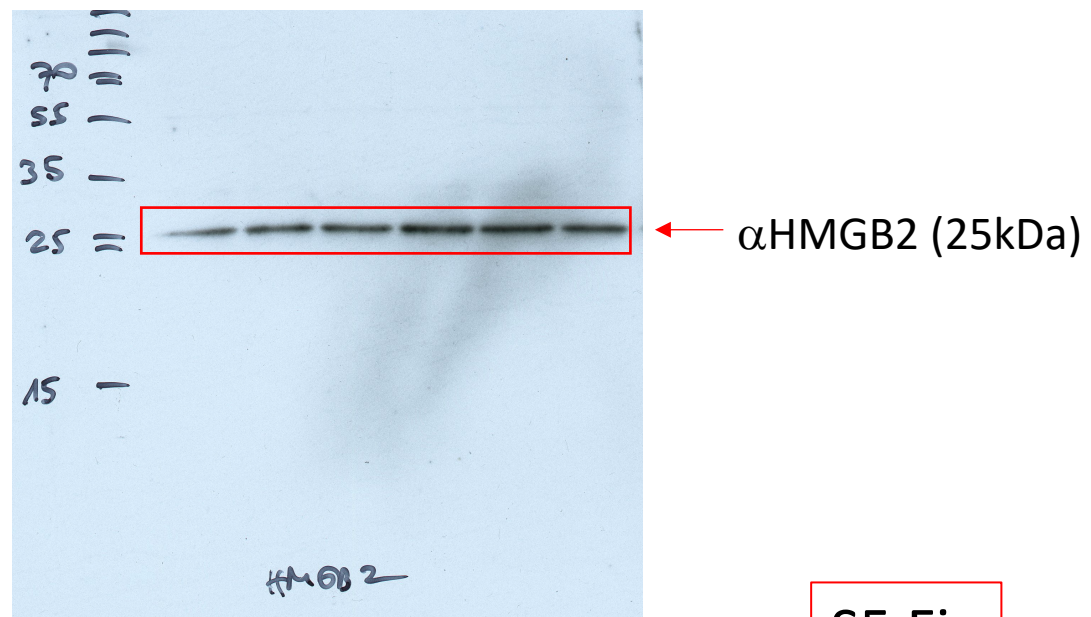

S5 Fig

Supplement: S13 Fig — The number of the corresponding Figure in the manuscript is indicated below the image (red rectangle). (PDF) [file pone.0233394.s013.pdf]
