# Supplementary material for: Gene expression regulation by the Chromodomain helicase DNA-binding protein 9 (CHD9) chromatin remodeler is dispensable for murine development
Source: PLoS One. 2020 May 26;15(5):e0233394. doi: 10.1371/journal.pone.0233394 (PMC7250415; doi:10.1371/journal.pone.0233394)

A. LIVER

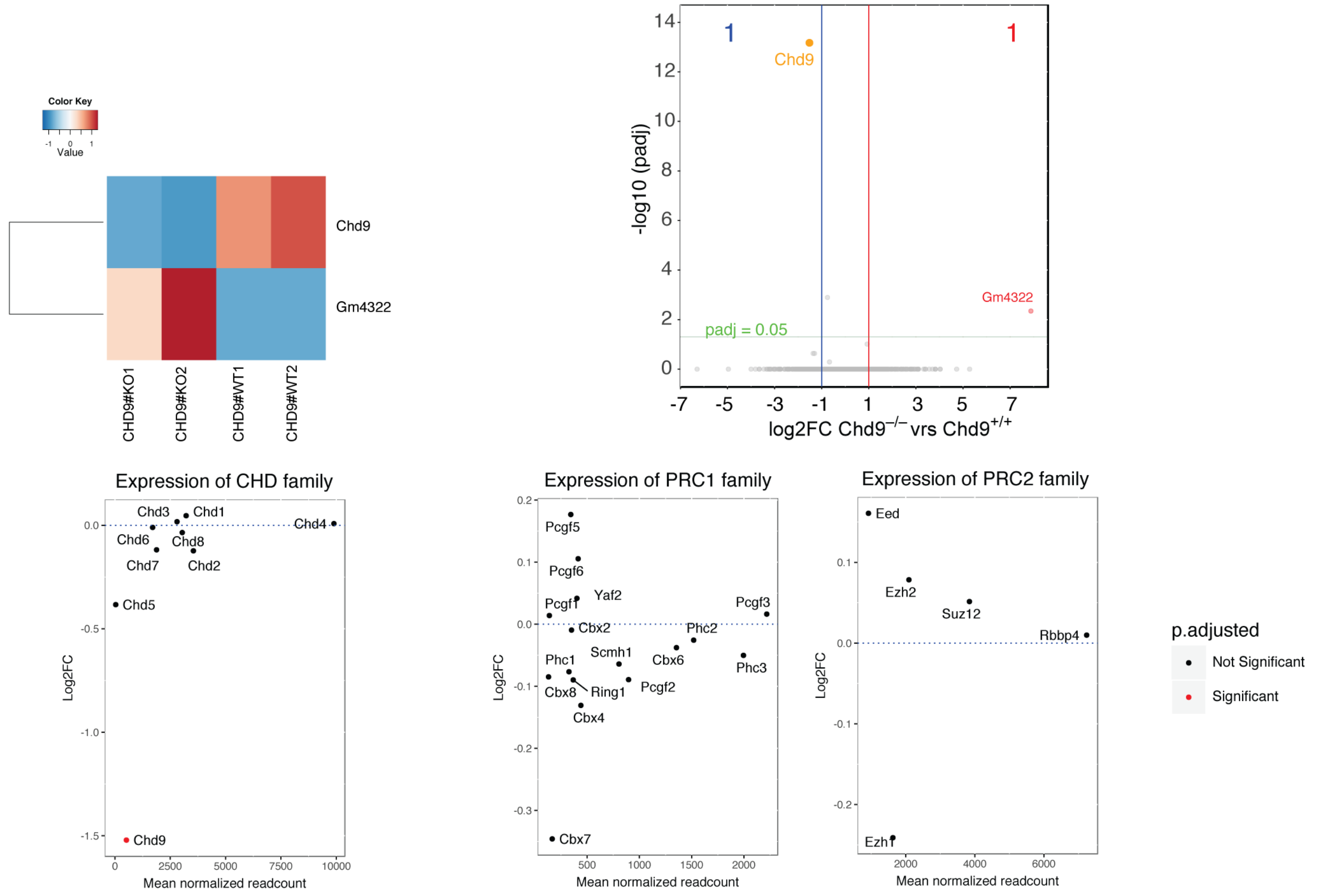

B. BRAIN

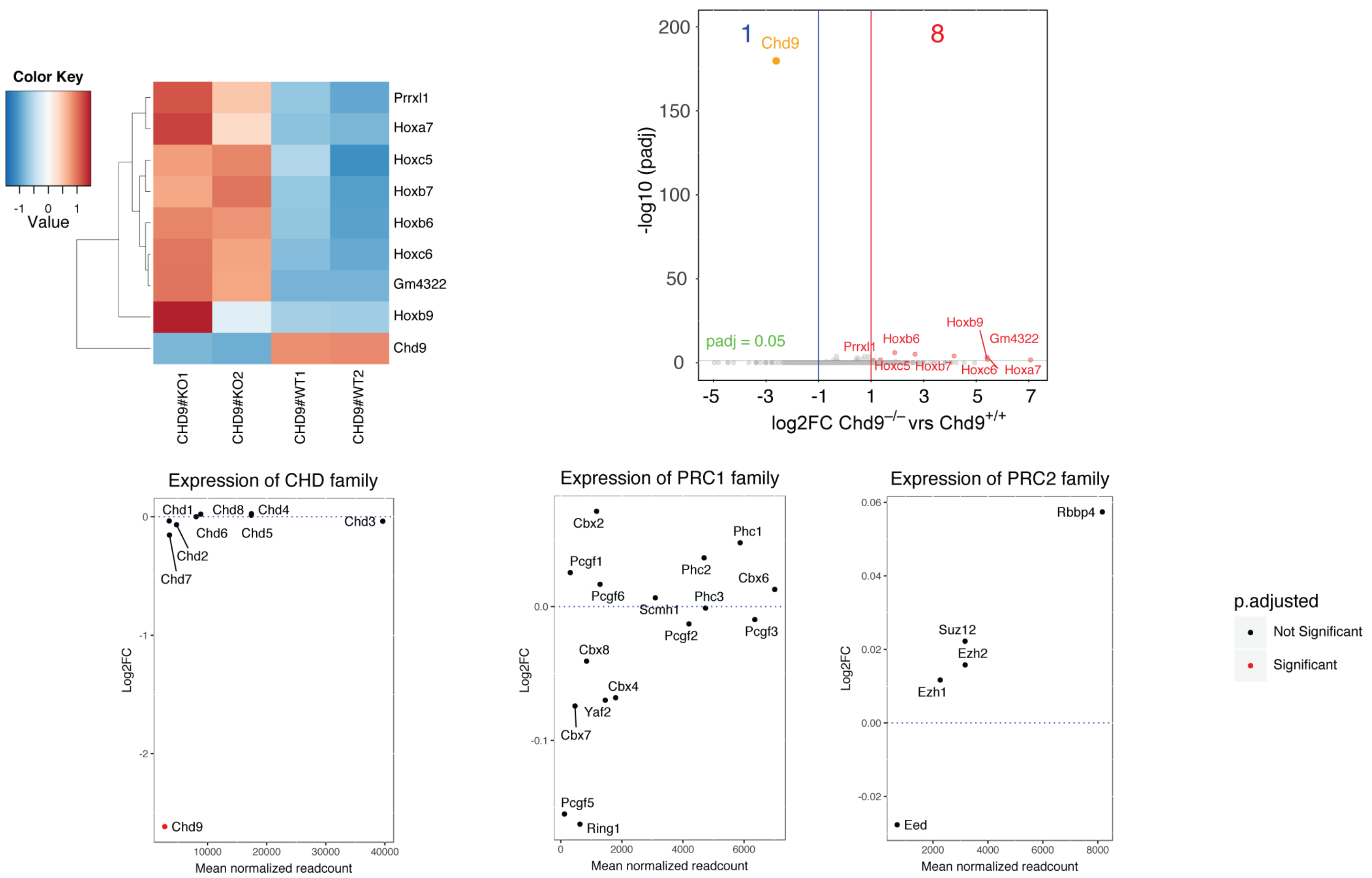

Supplement: S3 Fig — (A) Liver, (B) Brain (anticlockwise): Volcano plots represent differentially expressed genes (DEGs) between Chd9-/- and Chd9+/+ liver (A), brain (B). The DEGs with p.adj <0.05 and log2 (fold change) > ±1 are shown, Chd9 gene is highlighted in orange. Heatmap of statistically significant DEGs in Chd9-/- compared to control Chd9+/+ organs. The color scale is based on normalized read values. Scatter-plot expression profiles of each gene in the Polycomb repressive complex 1 (PRC1) and 2 (PRC2) and CHD family shown based on the log2 fold change over the average expression strength. Genes highlighted in red are found to be significant (p.adj < 0.05). (PDF) [file pone.0233394.s003.pdf]
