# Supplementary material for: Gene expression regulation by the Chromodomain helicase DNA-binding protein 9 (CHD9) chromatin remodeler is dispensable for murine development
Source: PLoS One. 2020 May 26;15(5):e0233394. doi: 10.1371/journal.pone.0233394 (PMC7250415; doi:10.1371/journal.pone.0233394)

A. K562

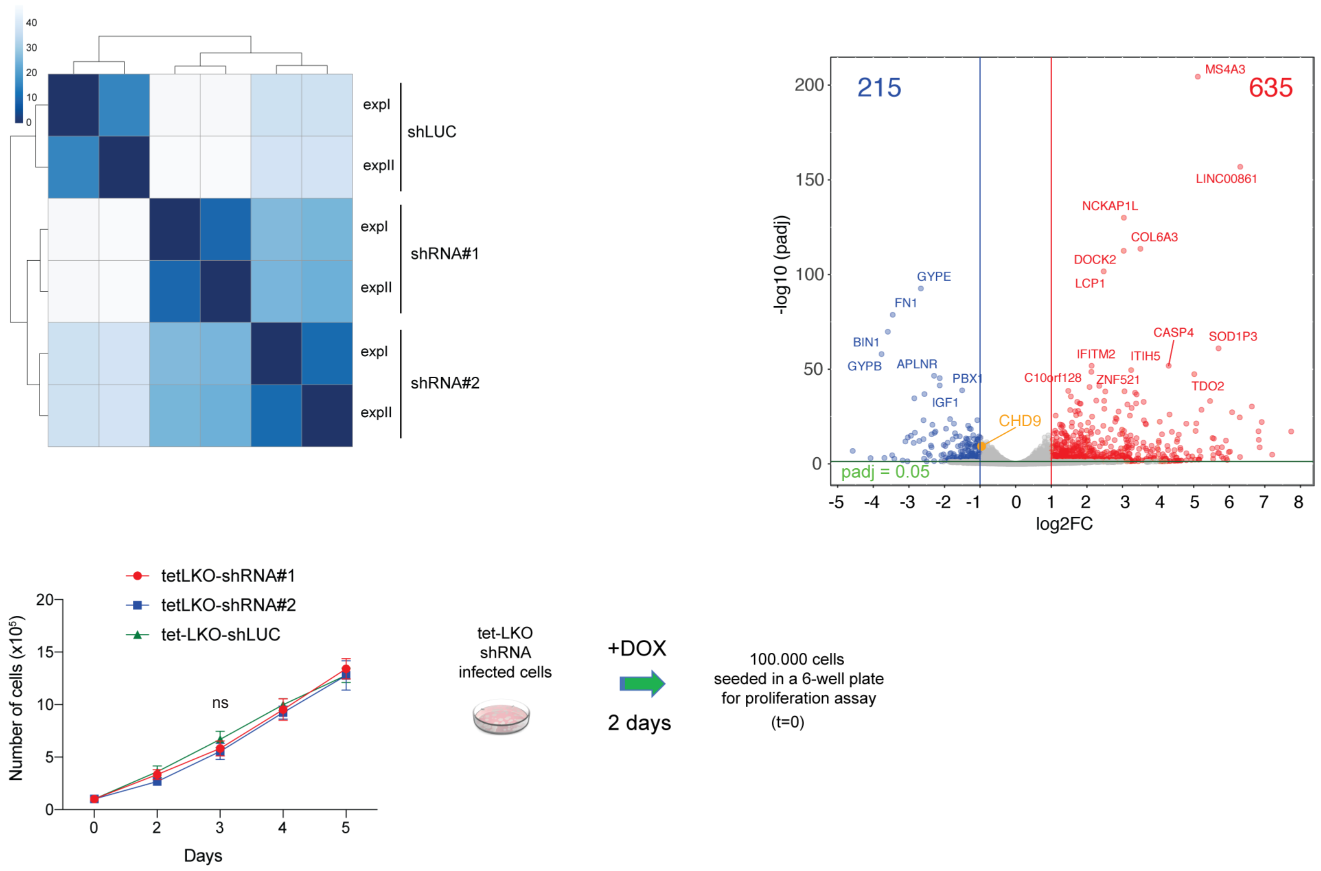

B. SNB19

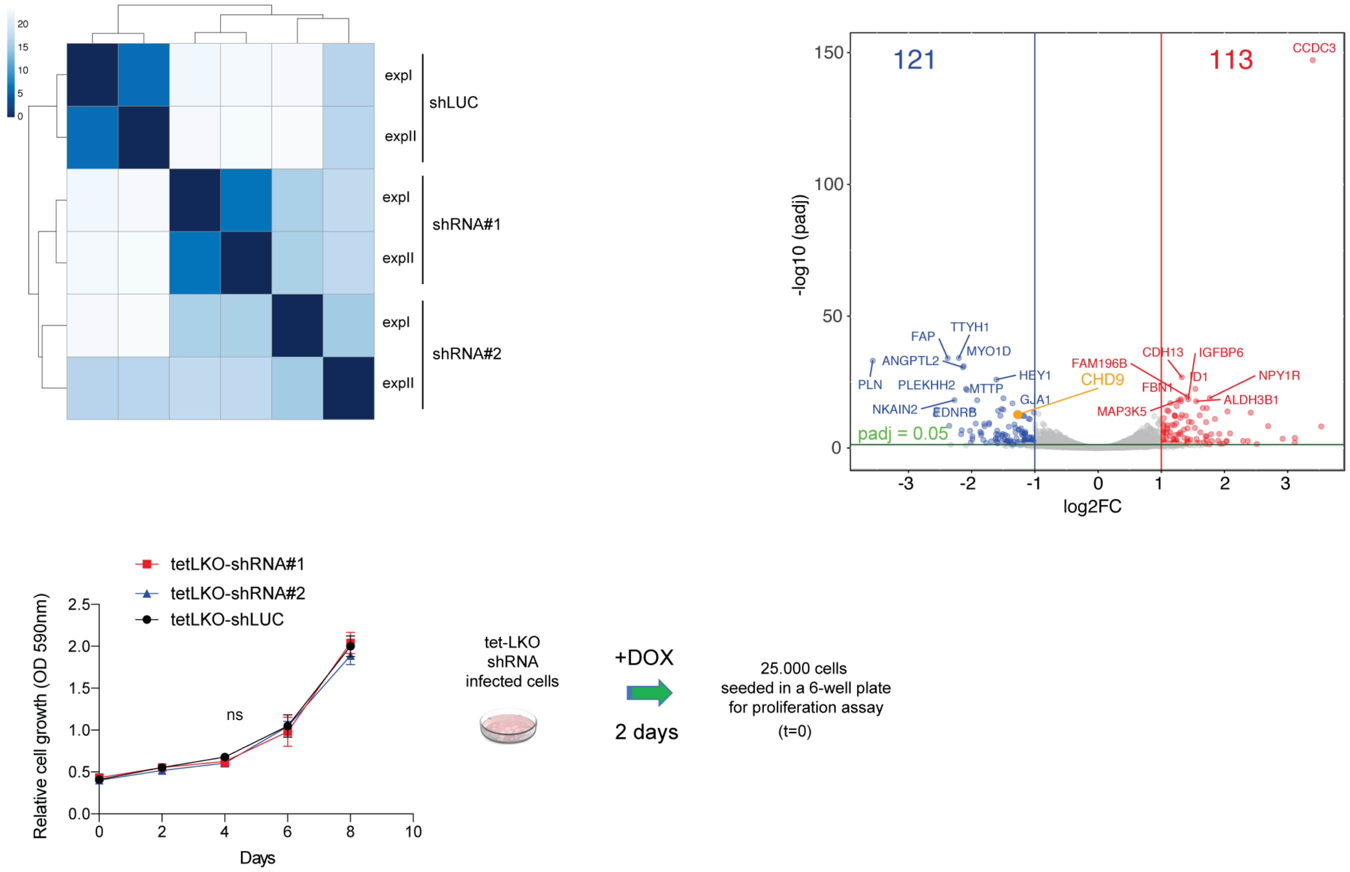

Supplement: S11 Fig — (A) K562 chronic myelogenous leukemia and (B) SNB19 glioblastoma: (left) Unsupervised hierarchical clustering of analyzed knockdown cells, each experiment was done in duplicate. (Right) Volcano plots represent significant differentially expressed genes (DEGs) between two shRNA hairpins targeting CHD9 and control hairpin targeting Luciferase. The DEGs with FDR < 0.05 and log2 (fold change) > ±1 are shown, 20 most significant genes are highlighted (down-blue, up-red); CHD9 gene is highlighted in orange. The DEGs are enumerated at the top of each plot. (Bellow) Proliferation curve representing relative growth of knockdown (shRNA#1 and shRNA#2 hairpins against CHD9) and control (Luciferase hairpin) cells. Cells were treated with 1μg/ml doxycycline (DOX) two days before seeding for proliferation assay. Doxycycline was kept throughout duration of the experiment. In case of K562, cell number was measured on cell counter, whereas for SNB19 glioblastoma relative cell growth was determined by crystal-violet assay. Data represented as average ± standard error mean (SEM), p-value was calculated using two-way ANOVA (for K562 cells p-value = 0.0902, for SNB19 cells p-value = 0.1977, ns = not significant). Cartoon illustrates seeding strategy. (PDF) [file pone.0233394.s011.pdf]
