# Supplementary material for: Gene expression regulation by the Chromodomain helicase DNA-binding protein 9 (CHD9) chromatin remodeler is dispensable for murine development
Source: PLoS One. 2020 May 26;15(5):e0233394. doi: 10.1371/journal.pone.0233394 (PMC7250415; doi:10.1371/journal.pone.0233394)
Supplement: S12 Fig — Each circle in the scatter plot represents a KEGG pathway, name of the pathway is denoted on the Y-axis and the enrichment score the X-axis. The size of the dots corresponds to the -log10(padj) value, indicating statistically significant KEGG categories. An enrichment score indicates over-representation (positive) or under-representation (negative) of the DEGs in a given pathway. KEGG categories mentioned in the main text are highlighted in red (over-represented), blue (under-represented). (PDF) [file pone.0233394.s012.pdf]

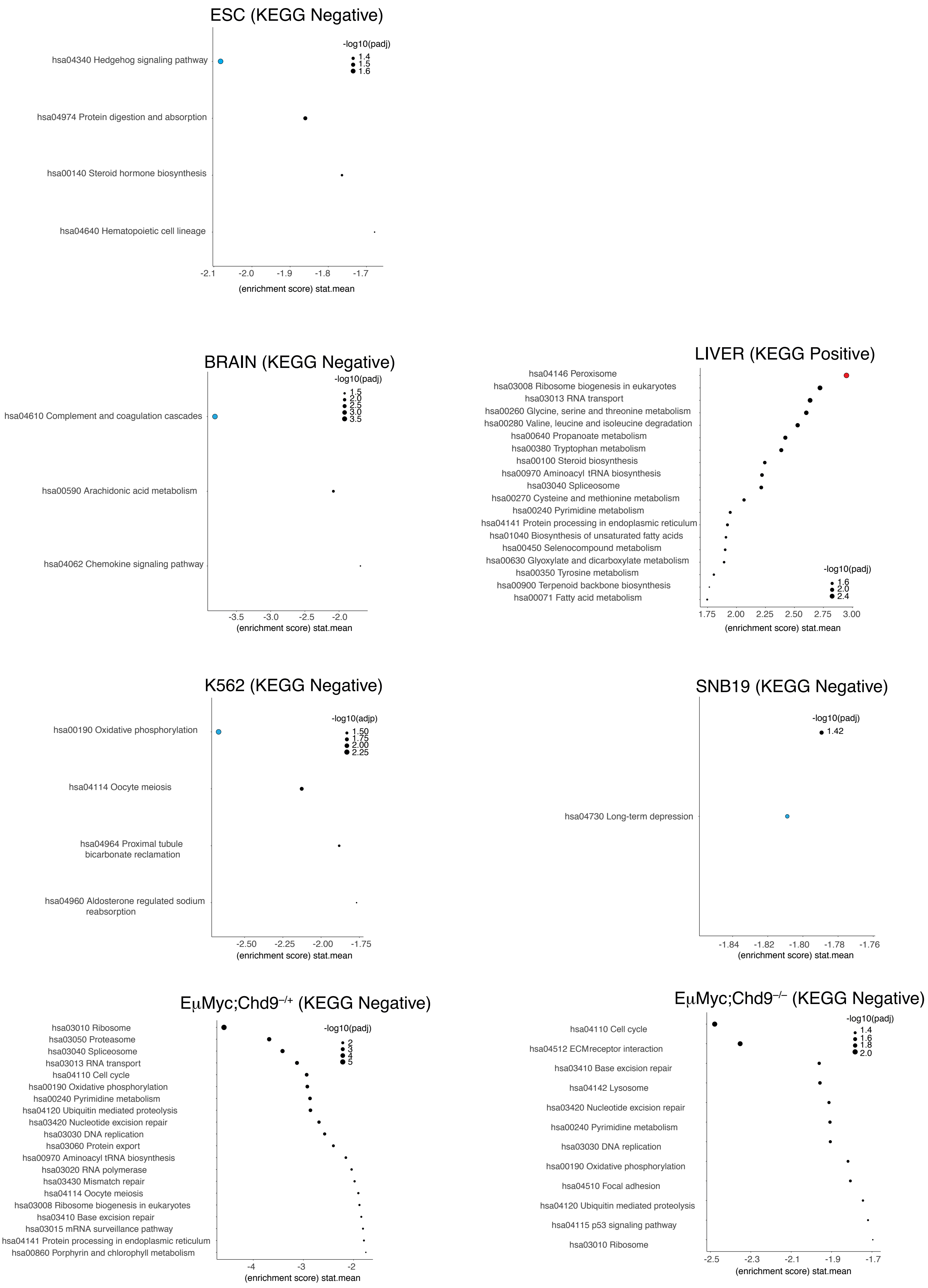

ESC (KEGG Negative)
-log10(padj)
1.4
1.5
1.6
hsa04340 Hedgehog signaling pathway
hsa04974 Protein digestion and absorption
hsa00140 Steroid hormone biosynthesis
hsa04640 Hematopoietic cell lineage
-2.1
-2.0
-1.9
-1.8
-1.7
(enrichment score) stat.mean
BRAIN (KEGG Negative)
-log10(padj)
1.5
2.0
2.5
3.0
3.5
hsa04610 Complement and coagulation cascades
hsa00590 Arachidonic acid metabolism
hsa04062 Chemokine signaling pathway
-3.5
-3.0
-2.5
-2.0
(enrichment score) stat.mean
LIVER (KEGG Positive)
hsa04146 Peroxisome
hsa03008 Ribosome biogenesis in eukaryotes
hsa03013 RNA transport
hsa00260 Glycine, serine and threonine metabolism
hsa00280 Valine, leucine and isoleucine degradation
hsa00640 Propanoate metabolism
hsa00380 Tryptophan metabolism
hsa00100 Steroid biosynthesis
hsa00970 Aminoacyl tRNA biosynthesis
hsa03040 Spliceosome
hsa00270 Cysteine and methionine metabolism
hsa00240 Pyrimidine metabolism
hsa04141 Protein processing in endoplasmic reticulum
hsa01040 Biosynthesis of unsaturated fatty acids
hsa00450 Selenocompound metabolism
hsa00630 Glyoxylate and dicarboxylate metabolism
hsa00350 Tyrosine metabolism
hsa00900 Terpenoid backbone biosynthesis
hsa00071 Fatty acid metabolism
-log10(padj)
1.6
2.0
2.4
1.75
2.00
2.25
2.50
2.75
3.00
(enrichment score) stat.mean
K562 (KEGG Negative)
-log10(adjp)
1.50
1.75
2.00
2.25
hsa00190 Oxidative phosphorylation
hsa04114 Oocyte meiosis
hsa04964 Proximal tubule bicarbonate reclamation
hsa04960 Aldosterone regulated sodium reabsorption
-2.50
-2.25
-2.00
-1.75
(enrichment score) stat.mean
SNB19 (KEGG Negative)
-log10(padj)
1.42
hsa04730 Long-term depression
-1.84
-1.82
-1.80
-1.78
-1.76
(enrichment score) stat.mean
EμMyc;Chd9–/+ (KEGG Negative)
-log10(padj)
2
3
4
5
hsa03010 Ribosome
hsa03050 Proteasome
hsa03040 Spliceosome
hsa03013 RNA transport
hsa04110 Cell cycle
hsa00190 Oxidative phosphorylation
hsa00240 Pyrimidine metabolism
hsa04120 Ubiquitin mediated proteolysis
hsa03420 Nucleotide excision repair
hsa03030 DNA replication
hsa03060 Protein export
hsa00970 Aminoacyl tRNA biosynthesis
hsa03020 RNA polymerase
hsa03430 Mismatch repair
hsa04114 Oocyte meiosis
hsa03008 Ribosome biogenesis in eukaryotes
hsa03410 Base excision repair
hsa03015 mRNA surveillance pathway
hsa04141 Protein processing in endoplasmic reticulum
hsa00860 Porphyrin and chlorophyll metabolism
-4
-3
-2
(enrichment score) stat.mean
EμMyc;Chd9–/– (KEGG Negative)
-log10(padj)
1.4
1.6
1.8
2.0
hsa04110 Cell cycle
hsa04512 ECMreceptor interaction
hsa03410 Base excision repair
hsa04142 Lysosome
hsa03420 Nucleotide excision repair
hsa00240 Pyrimidine metabolism
hsa03030 DNA replication
hsa00190 Oxidative phosphorylation
hsa04510 Focal adhesion
hsa04120 Ubiquitin mediated proteolysis
hsa04115 p53 signaling pathway
hsa03010 Ribosome
-2.5
-2.3
-2.1
-1.9
-1.7
(enrichment score) stat.mean
